# One-Step Photocatalytic Oxidation of Cumene with Mesoporous Carbon Nitride

**DOI:** 10.1021/acscatal.5c08511

**Published:** 2026-03-02

**Authors:** Sonia Żółtowska, Gabriel Chan, Tim Tjardts, Paolo Giusto

**Affiliations:** † Department of Colloid Chemistry, 28321 Max Planck Institute of Colloids and Interfaces, 14476 Potsdam, Germany; ‡ Institute of Material Science, 9179 Christian-Albrechts-Universität zu Kiel, 24143 Kiel, Germany; § Queensland University of Technology, School of Chemistry and Physics, Joint Laboratory on Nanocatalysis for Sustainable Chemistry, Brisbane, Queensland 4000, Australia

**Keywords:** carbon nitride, photocatalysis, metal-free catalyst, benzylic oxidation, cumene, scale-up

## Abstract

The shift toward mild, low-impact chemical conversion is pivotal for future development. Photocatalytic energy conversion, which harnesses light to drive reactions under mild conditions, offers a powerful alternative to conventional energy-intensive synthetic routes. Here, we establish mesoporous graphitic carbon nitride (mpg-CN) as a highly efficient, metal-free photocatalyst for the selective oxidation of benzylic substrates, using cumene as a representative example. Optimized conditions enabled the selective oxidation of cumene to acetophenone and 1-methoxyethylbenzene with high efficiency, underscoring the catalyst’s reaction selectivity and synthetic value. The system readily accommodates structurally related substrates with radical stability and steric hindrance playing defining roles in reactivity. Sensitivity analyses revealed critical parameters governing reaction performance, while spin-trapping experiments under oxygen-rich conditions identified hydroperoxide radicals as key intermediates initiating α-carbon oxidation. Notably, scaling the reaction 10-fold preserved 66% conversion and 70% selectivity, and a further scale-up to gram scale of precursor delivered 50% acetophenone after 72 h. Furthemore, solvent recovery and reuse maintained high catalytic efficiency over the initial reuse cycles, demonstrating the operational durability of the system. Collectively, these findings position mpg-CN as a robust, scalable, and metal-free photocatalyst, a compelling platform for next-generation chemical manufacturing.

## Graphical abstract

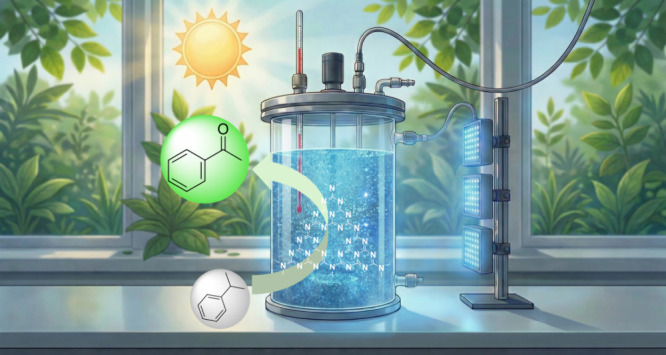

## 1 Introduction

In the last decades, the need for alternative synthesis solutions has significantly increased due to the pressing global challenges associated with energy consumption and environmental exploitation.^1^ Industrial chemical processes are usually energy intensive and among the largest contributors to carbon dioxide emissions,^2^ requiring a shift toward low-carbon or even carbon-neutral strategies. Scientists worldwide are actively working to develop sustainable energy conversion solutions that consider the entire lifecycle of a reaction—from synthesis to waste utilization—minimizing the environmental impact.^3^

Among these solutions, visible light photocatalysis has emerged as a powerful tool in modern organic synthesis. With solar energy as a renewable input, selective transformations are performed under mild reaction conditions, significantly reducing energy consumption. Homogeneous photocatalytic systems typically consist of organic dyes and transition metal complexes which show high quantum efficiency and extended excited-state lifetimes, making them highly favorable photocatalysts.^4–9^ However, these systems often suffer from high costs, lower stability, and multiple recovery steps for catalyst separation and reuse, hindering further incorporation into practical industrial use-cases.

Therefore, researcher’s focus in the recent years has shifted toward metal-free heterogeneous photocatalysis, which offers greater stability, simple separation, and high reusability, as a more practical alternative for large-scale applications.^10–13^ Unlike homogeneous catalyst, where catalyst decomposition and photobleaching is still a major challenge,^14–16^ the heterogeneous ones offer high chemical and thermal stability and can be quantitatively recovered after the chemical conversion process. Among those, mesoporous graphitic carbon nitride (mpg-CN) is a versatile and metal-free material with excellent photocatalytic activity for both oxidative and reductive light-induced transformations.^17,18^ mpg-CN is a metal-free semiconductor which is obtained by the thermal condensation of N-rich organic precursors in the presence of a hard templating agent. Its medium band gap of 2.7 eV enables efficient visible-light absorption, making it well-suited for visible light-driven transformations: in fact, mpg-CN has demonstrated remarkable versatility, showing high catalytic activity for a broad spectrum of oxidation and reduction reactions, including benzylic C–H oxidation, C–N coupling reactions, and hydrogen evolution reactions, while maintaining excellent stability and reusability.^19–24^ Furthermore, the templating process provides it with a high surface area of 100 – 250 m^2^ g^–1^, increasing the amount of available active sites for the reactions. The wide availability of the precursors, the electronic and structural properties make mpg-CN an attractive candidate as heterogeneous photocatalyst.^18,25,26^ Despite its advantages and the broad utilization of mpg-CN for organic reactions, its application for gram-scale photocatalytic reactions is still a major challenge, primarily due to mass transfer limitations, homogeneous distribution of the catalyst, and reduced light penetration in larger reaction volumes.^13,27,28^ Overcoming these barriers is crucial for its practical application in industrial-scale green chemistry processes.

Cumene is a widely used industrial precursor for the synthesis of phenol via the Hock process due to its low cost and industrial availability. The synthesis proceeds via the formation of the cumene hydroperoxide (CHP) intermediate, followed by an acid-catalyzed step to convert it to phenol and acetone as a byproduct. However, in the second step, the hydroperoxide intermediate can be converted to acetophenone (and methanol) under alkaline conditions. In all cases, the reaction involves the use of high pressure of O_2_ (5–7 bar) and relatively high temperatures (<100 °C) for the formation of the hydroperoxide intermediate. In the second step, typically strong acids (sulfuric acid) or strong bases (metal hydroxides) are used for the final conversion of the hydroperoxide.^29–35^ The harsh conditions used for the conversion of cumene require special equipment and the downstream treatment of large volumes of acid/base solutions. For these reasons, cumene oxidation is an ideal case study for photocatalytic conversion to overcome these critical issues using a widely available and industrially relevant precursor. Given its industrial significance, cumene oxidation serves as a benchmark reaction for investigating selectivity, radical pathways, and alternative oxidation mechanisms in photocatalysis. In particular, there is a growing demand for carbonyl compounds, which are among the most important building blocks for pharmaceuticals, agrochemicals, and polymer synthesis. Therefore, the development of a one-step efficient, scalable, and in mild conditions synthesis of carbonyl compounds from industrially relevant sources is of significant interest.^36,37^

This study presents a simple, sustainable, and metal-free photocatalytic method for the selective aerobic oxidation of cumene at the benzylic position, yielding acetophenone with a high efficiency under ambient conditions. In contrast to conventional benzylic oxidation protocols that require on elevated temperatures, transition metal catalysts (e.g., Mn, Cu, or Co complexes), or pressurized O_2_ environments,^11,38–40^ this method operates at room temperature, standard pressure, and in mild reaction conditions, thereby reducing energy consumption and increasing atom economy. The system relies solely on molecular oxygen as the oxidant and also proceeds even in the absence of an additional oxygen supply, further demonstrating its efficiency and environmental benefits. Furthemore, a key objective of this study is to systematically evaluate reaction conditions providing a sensitivity assessment to achieve high efficiency and selectivity across diverse substrates.^41^ To demonstrate the system’s versatility, we applied it to a broad spectrum of alkyl-substituted aromatic derivatives, revealing consistently high selectivity and conversion across diverse substrates. Mechanistic investigations using spin-trapping EPR spectroscopy revealed hydroperoxide radicals as key intermediates in the α-C–H oxidation pathway, providing evidence supporting the proton coupled electron transfer mechanism. These findings, combined with the photocatalyst’s reusability over multiple cycles with low loss of activity, establish mpg-CN as a durable, low-cost, and environmentally benign platform with potential for scale-up.

To evaluate the system’s scalability, we conducted oxidation of cumene on a gram scale, achieving 50% isolated yield of acetophenone under ambient conditions, demonstrating the method’s operational viability beyond conventional bench-scale experiments. Given that cumene is already a key substrate in industrial chemical production, integrating this visible-light-driven, metal-free photocatalytic protocol into industrial oxidation workflows could significantly reduce energy input, eliminate transition metal waste streams, and streamline the production of benzylic ketones. The adoption of mpg-CN photocatalysis in existing chemical manufacturing platforms offers a concrete route toward greener oxidation processes beyond the mg-scale.

## 2 Results and Discussion

Inspired by the cumene process, we initially selected cumene as a model substrate for the oxidation reactions. Unlike the traditional industrial method, which typically requires elevated temperatures and pressures of O_2_, our approach employs mild, liquid-phase conditions at room temperature, eliminating the need for excess oxidants or extreme reaction parameters. This not only enhances environmental sustainability but also aligns with the principles of sustainable chemistry, for example, reducing energy consumption and minimizing hazardous byproducts. Under our standard reaction conditions (Table 1), we observed an unexpected product distribution. Instead of the conventional formation of acetone and phenol, the major products were acetophenone (1) and 1-methoxyethylbenzene (2), with a high selectivity toward acetophenone.

**1:**
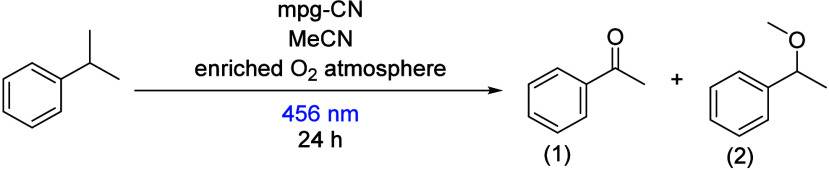
Overall Reaction Scheme of Cumene Oxidation

**Table 1:** Optimization of the Reaction Conditions^a^

Entry	Deviation from standard conditions	Conversion of substrate^b^	Selectivity for (1)^b^
1	N/A	81%	79%
2	30 mg mpg-CN	57%	76%
3	10 mg mpg-CN	5%	74%
4	Ambient air	37%	75%
5	N_2_	N/A	N/A
6	Ethyl acetate	27%	46%
7	500 nm LED	traces	N/A
8	Dark	N/A	N/A

a Standard reaction conditions: Cumene (0.05 mmol), mpg-CN (20 mg), acetonitrile (3 mL), blue LEDs (λ_max_ = 456 nm, 80 mW cm^–2^), 25 °C, 24 h. N/A – not detected.

b measured using GC/MS

To identify the parameters governing selectivity and yield in the photocatalytic oxidation of cumene, we systematically varied catalyst loading, oxygen availability, irradiation wavelength, and solvent properties (Table 1, Entries 2–8). By fine-tuning these parameters, we aimed to identify the optimal conditions that maximize conversion and selectivity of the reaction, providing deeper insights into the underlying photocatalytic mechanism. The best catalyst loading of 20 mg of mpg-CN was identified for this reaction scale. Halving the amount to 10 mg drastically reduced conversion to 5%, while increasing the amount to 30 mg decreased conversion to 57%, likely due to excessive light scattering and lower light penetration, which limits effective photoexcitation. The role of the oxidant in the reaction mechanism was also examined. When the reaction was conducted in ambient air (Entry 4), some conversion (37%) was still observed, and selectivity toward acetophenone remained high (75%). However, in the absence of oxygen (Entry 5, under N_2_ atmosphere), no conversion was detected, confirming that oxygen plays a crucial role. These findings support a mechanism involving photogenerated reactive oxygen species (ROS), such as superoxide (O_2_^•–^) and hydroxyl (^•^OH) radicals, formed upon oxygen activation by photoexcited mpg-CN (for comparison see Figure 1). The effect of the solvent on the reaction efficiency was also investigated. The reduced conversion observed in ethyl acetate (27%) compared to acetonitrile is attributed to its lower oxygen solubility, weaker oxygen-solvent interactions, and reduced polarity,.^42,43^ Solvent effects play a critical role in photocatalytic oxidation reactions by influencing charge carrier dynamics and reactive oxygen species (ROS) chemistry. Acetonitrile possesses a higher dielectric constant (ε ≈ 42.3) compared to ethyl acetate (ε ≈ 6) (See supplementary note 2), which can enhance the stabilization of photogenerated charge carriers and reduce recombination rates.^44^ Moreover, acetonitrile is reported to dissolve concentrations of molecular oxygen that are higher than those of ethyl acetate, increasing the availability of O_2_ for reduction by photogenerated electrons and subsequent ROS formation. These combined effects are likely responsible for the higher activity observed in acetonitrile under the otherwise same photocatalytic conditions. Control experiments under 500 nm LED light and in the absence of light (Entry 7 and 8) yielded no conversion, confirming that photoexcitation of mpg-CN is essential to initiate the reaction.

**Figure 1:**
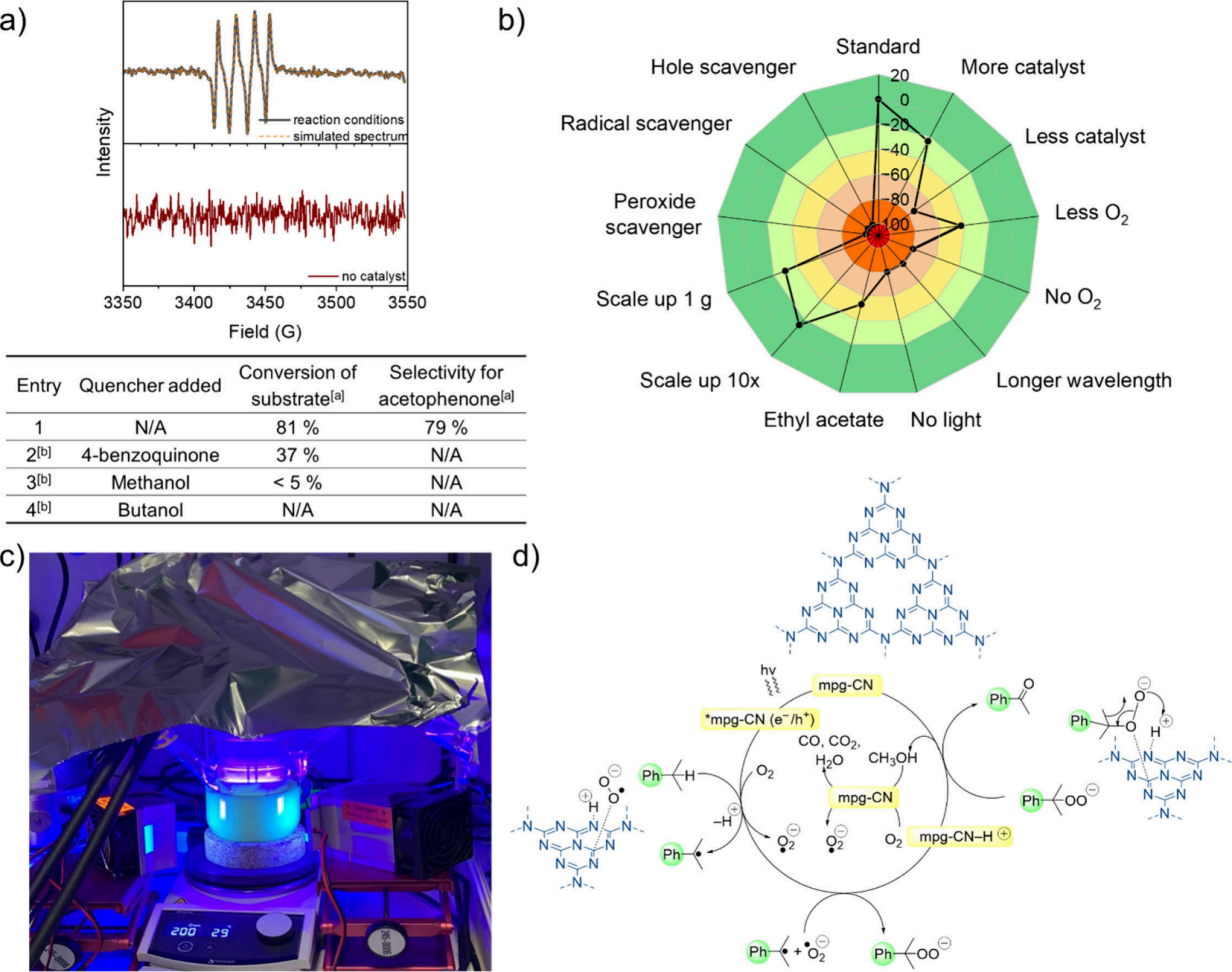
a) Experimental and simulated spectra of DMPO spin adducts of the superoxide anion (O_2_^•–^) in reaction conditions (upper) and without catalyst (lower) wth scavengers applied for photocatalytic tests: standard reaction conditions: cumene (0.005 mmol), mpg-CN (20 mg), acetonitrile (3 mL), three blue LEDs (λ_max_ = 456 nm, 80 mW cm^–2^), 25 °C, 24 h. N/A – not detected; ^[a]^ measured using GC/MS; ^[b]^ 25 mmol of the quencher was added; b) effects of alternating reaction conditions to yield of acetophenone, visualized by a radar diagram; c) customized photoreactor for 1 g scale of cumene oxidation; d) proposed mechanism of cumene oxidation.

To probe the substrate scope of the mpg-CN photocatalytic system, we examined how benzylic substituents influence the oxidation efficiency and selectivity. Understanding how different functional groups impact conversion and selectivity is key to optimizing the system for broader applications in green synthesis. Arenes structurally related to cumene, including toluene, ethylbenzene, p-cymene, and 4-ethyltoluene, were selected to evaluate how benzylic substitution patterns affect radical stability, conversion, and selectivity (Scheme 2). Under the standard reaction conditions, all three compounds exhibited good conversion and selectivity. However, toluene itself showed a relatively low conversion of only 50%, despite maintaining high selectivity toward the carbonyl product. The lower conversion of toluene is attributed to the lower stabilization of the intermediate nonsubstituted benzyl radical formed during oxidation resulting in a higher bond dissociation free energy (BDFE, 364 kJ/mol) for its benzylic C–H bond compared to, for example, ethylbenzene (∼301 kJ/mol).^45–47^ This higher BDFE in toluene means that the bond is more stable against cleavage, leading to lower conversion rates during the oxidation process. Radical stability plays a central role in determining the oxidation efficiency. Benzyl radicals derived from ethylbenzene and *p-*ethyltoluene are secondary and benefit from greater hyperconjugation, in contrast to the primary benzyl radical from toluene. Additionally, electron-donating alkyl substitutents increase radical stability via inductive effects, further enhancing intermiediate stability. The lower reactivity of the tertiary C­(sp^3^)–H benzylic group is instead attributed to the cleavage of a benzylic C­(sp^3^)–C­(sp^3^) bond. While the BDFE for benzylic C­(sp^3^)–H is lower in cumene than in ethylbenzene, the cleavage of the alkyl C­(sp^3^)–C­(sp^3^) bonds is more challenging. Indeed, in contrast to what observed by Leibler et al.^48^ for the selective phototocatalytic monofluorination of series of benzylic molecules consistent with relative BDFE (tertiary > secondary > primary) via hydrogen atom transfer (HAT). On the other hand, we observe a more favorable conversion for secondary > tertiary > primary, inferring that cleavage of the C­(sp^3^)–C­(sp^3^) is more difficult than that of the C­(sp^3^)–H. The C­(sp^3^)–C­(sp^3^) bond cleavage by means of carbon nitride materials has been observed in the presence of strong oxidating agents, such as persulfate, or at higher treatment temperature, such as for the degradation of polystyrene, and attributed in all cases to the presence of O_2_^•–^.^49,50^

**Scheme 2:**
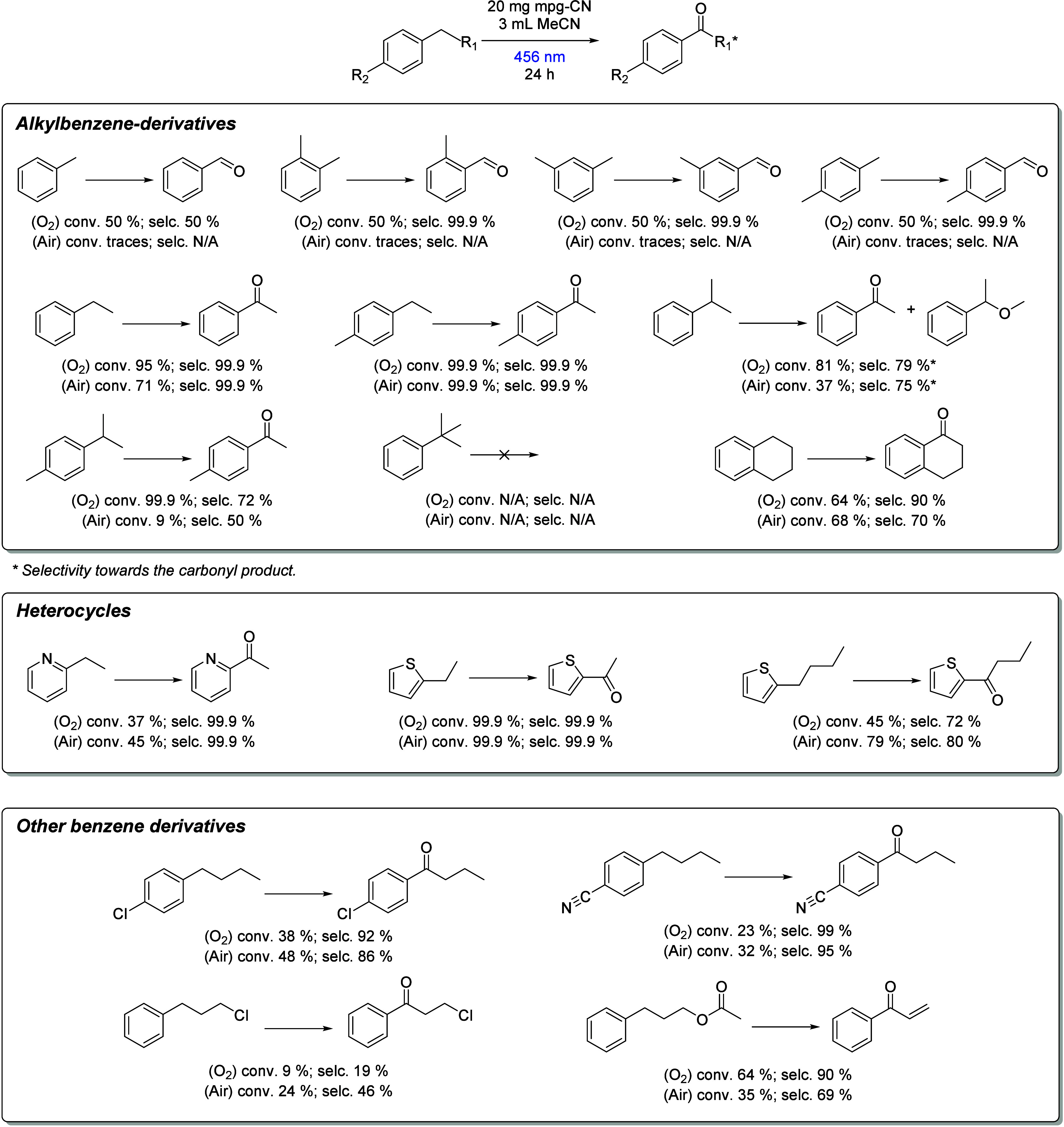
Scope of the Reaction^a^

a Standard reaction conditions: substrate (0.05 mmol), mpg-CN (20 mg), acetonitrile (3 mL), blue LEDs (λ_max_ = 456 nm, 80 mW cm^–2^), 25 °C, 24 h. N/A – not detected.

To further probe the versatility of the mpg-CN photocatalytic system, we extended the substrate scope to include halogenated and heterocyclic aromatic compounds, which typically present greater challenges in radical-mediated oxidations. Most halogenated and heterocyclic substrates displayed moderate conversion rates (∼40%), with notable exceptions being 2-ethylthiophene (99%) and 3-phenylpropyl acetate (64%), indicating enhanced reactivity under the standard conditions. This can be attributed to a combination of electronic and steric effects. Halogen substituents exert strong inductive electron-withdrawing effects that destabilize the benzylic radical intermediate, thereby hindering hydrogen abstraction and lowering the overall conversion efficiency. In heteroaromatic substrates, lone pairs on heteroatoms such as sulfur or nitrogen can delocalize into the π-system, stabilizing the aromatic framework and making homolytic cleavage of adjacent C–H bonds less favorable for radical formation.^51^ Steric hindrance from bulky substituents can further impede radical propagation, thereby reducing conversion efficiency. The high reactivity of 2-ethylthiophene may arise from the enhanced delocalization of the spin density through the sulfur atom and adjacent π-system, facilitating radical stabilization. In the case of 3-phenylpropyl acetate, the presence of an electron-withdrawing ester group and a benzylic activation site enhances the formation and persistence of reactive intermediates.

Replacing the oxygen-enriched atmosphere with ambient air led to a substantial decrease in conversion, confirming that elevated oxygen levels are critical for efficient radical initiation and oxidation. However, two exceptions, ethylbenzene and 4-ethyltoluene, maintained high conversion and selectivity, even under ambient air conditions. This behavior is attributed to the formation of relatively stable secondary benzylic radicals in both substrates, which persist long enough to undergo efficient oxygen trapping and subsequent oxidation, even under ambient oxygen levels. The oxidation mechanism of ethylbenzene typically involves the formation of hydroperoxyl radicals and the subsequent abstraction of hydrogen atoms from the benzylic position. This pathway is facilitated by the presence of oxygen, even at lower concentrations found in ambient air.^52^ Similarly, 4-ethyltoluene undergoes oxidation to yield products that are more readily formed under ambient conditions, maintaining higher conversion rates. Several halogenated and heteroaromatic substrates showed improved conversion under ambient air, potentially due to the suppression of overoxidation or radical quenching pathways that are more prevalent in oxygen-rich environments.

A crucial aspect of this study focused on investigating the oxidation mechanism using cumene as a model substrate. To examine the formation of radical species, electron paramagnetic resonance (EPR) spectroscopy was performed in oxygen-saturated acetonitrile, employing 5,5-dimethyl-1-pyrroline-N-oxide (DMPO) as a spin trap (Figure 1A).

The EPR spectrum obtained under the reaction conditions displays a characteristic four-line hyperfine splitting pattern, which aligns with the expected spin-trapped hydroperoxide radicals. The strong correlation between the experimental spectrum and the simulated profile further confirms it. Moreover, the absence of a signal in the catalyst-free system indicates that mpg-CN is crucial for oxygen radical anion generation, through the generation of electron/hole pairs upon light absorption. Additionally, to determine the dominant reactive species, scavenger tests were conducted, as summarized in Figure 1A. In heterogeneous photocatalysis, efficient electron–hole separation is crucial for driving redox reactions. Scavenger tests further elucidate the role of reactive oxygen species in the oxidation selectivity. The addition of 4-benzoquinone, a hydroperoxide scavenger, shifted the main product to 1-methoxyethylbenzene, indicating that hydroperoxyl radicals (^•^OOH) are directly involved in acetophenone formation.^53^ Interestingly, when butanol (a radical scavenger) was introduced, only traces of butanoic acid were formed, indicating a shift away from the typical cumene oxidation pathway. This suggests that radical-mediated oxidation, with the formation of oxygen radical anion (and cumyl hydroperoxide), is the dominant mechanism in the absence of radical scavengers.^54^ The role of photogenerated holes in oxidation was examined by using methanol as a hole scavenger. Under these conditions, the formation of acetophenone was significantly suppressed, suggesting that holes initiate the oxidation of cumene by generating the cumyl radical cation, which subsequently reacts with molecular oxygen to form the peroxo intermediate. By scavenging holes, methanol reduces the overall conversion while still allowing for minor oxidative pathways.

The stability of the radical intermediates plays a crucial role in determining the efficiency of the oxidation process. The cumyl radical is stabilized by resonance, allowing it to persist long enough to react with oxygen and form a peroxy radical. However, competing termination pathways, such as radical recombination or solvent quenching, may limit the overall conversion efficiency. The presence of hydroperoxyl radicals supports the hypothesis that oxidation proceeds via an oxygen-involved radical chain pathway, rather than by exclusive direct electron transfer.^54^

To summarize the effects of varying reaction conditions to the yield of acetophenone from cumene, a color-coded radar diagram, inspired by Glorius and co-workers,^55^ was utilized to visualize the results (Figure 1B). The radar diagram clearly demonstrates that the standard reaction conditions yield the highest conversion in acetophenone. The reaction relies on light irradiation with wavelengths shorter than 500 nm and the presence of oxygen. The addition of radical (butanol), hole (metanol), and peroxide (4-benzoquinone) scavengers in the reaction mixture suppressed acetophenone formation entirely, confirming the essential role of photogenerated holes and radical intermediates in the oxidation process.

Despite significant advances in photocatalysis, scaling up reactions to practical levels remains a major challenge, particularly for carbon-nitride-based systems, where most reported photocatalytic transformations have been conducted at the micromolar scale. Scaling up cumene oxidation with mpg-CN is essential to evaluate larger scale applicability, identify scale-dependent limitations, and ensure reaction reproducibility under intensified conditions. At larger reaction scales, several factors influence photocatalytic performance, such as reduced photon flux due to limited light penetration, oxygen mass transfer, and inefficient heat dissipation, all of which can affect photoexcitation, reaction stability, and product selectivity. For example, oxygen dissolution and diffusion at the catalyst surface are critical for the formation of the oxygen radical anion, ultimately lowering conversion rates and selectivity. To systematically investigate scalability, the reaction was first scaled up 10-fold (from 50 μmol to 0.5 mmol of cumene) under identical conditions, yielding 66% conversion and 70% selectivity toward acetophenone after 24 h, a promising result that demonstrates partial transferability of the optimized conditions. Building on these results, we further scaled up to 1 g of cumene in a custom-built photoreactor (see Figure 1C), adjusting oxygen supply and catalyst loading to match increased reaction volume. However, at the gram scale, conversion dropped below 10% after 24 h, likely due to the lower light penetration and decreased photon flux per catalyst surface area, resulting in a reduced efficiency in reactive oxygen species generation. To compensate for these effects, the reaction was extended to 72 h, yielding 0.46 g of acetophenone (51%), that can be extracted quantitatively after simple column chromatography (see Materials and Methods for details). Further implementation into potential gram-scale conversion has been shown by Zhu et al, demonstrating an efficient cumene oxidation in a fixed-bed reactor with short residence times, however, with higher selectivity only to cumene hydroperoxide which is only the first step of the reaction.^56^ On the other hand, our system provides a suitable strategy for the one-step g-scale conversion in batch systems, overcoming the drawbacks of multistep reaction with high selectivity toward acetophenone. We envision that this work provides a solid foundation for further practical implementation, including future translation to continuous-flow operations potentially relevant to industrial settings.

Based on the above findings, a mechanistic pathway for the mpg-CN-catalyzed oxidation of cumene was proposed (Figure 1D). Upon light irradiation, mpg-CN undergoes photoexcitation, generating an electron–hole pair that separates into mobile charge carriers. The valence-band hole abstracts a hydrogen atom from the benzylic position of cumene via PCET mechanism,^57^ forming a cumyl radical. At the same time, the excited electron reduces molecular oxygen to a superoxide radical (O_2_^•–^). The spatial proximity between the surface-bound superoxide and the cumyl radical facilitates rapid radical–radical coupling, yielding a cumylperoxide intermediate. In a second photocatalytic step, this intermediate takes up the proton from mpg-CN–H^+^ and undergoes pericyclic reaction, cleaving the adjacent C–C bond to produce acetophenone and methanol, by coupling the methyl group with the hydroxy group generated from the peroxide group. The methanol evolved is quickly oxidized in the reaction conditions to CO/CO_2_ (and water) (See supplementary note 9).^58–61^ The rapid conversion of methanol to CO and CO_2_ is expected since methanol is widely used as a sacrificial agent, i.e., for hydrogen evolution reactions (HER), taking up the photogenerated holes at the valence band. For this reason cannot be directly detected in the current setup. Indeed, as previously shown, when using methanol as a scavenger it hinders the formation of the cumyl radical, suppressing the proceeding of the reaction. Additionally, the mechanism explains the formation of a methoxy radical bound to the surface of mpg-CN during the methanol oxidation step, leading to the evolution of methylperoxide, thermally decomposing in smaller products such as water and formaldehyde (Figure S10).

The challenges of the 21st century drive the scientific community not only to focus on the activity of proposed catalytic systems but also to carefully assess the potential for reusing key components of the photocatalytic system, such as the photocatalyst and solvent. A detailed investigation into catalyst reusability and deactivation pathways is presented in Supplementary Note 2. While the catalyst retained considerable activity under both ambient air and oxygen-rich conditions, a moderate decrease in conversion across successive cycles suggests some changes to surface activity, potentially due to mild surface oxidation or alteration of active sites. Optical, structural, and spectroscopic analyses of the used catalyst indicated minor modifications such as slight band gap shifts, increased surface hydroxylation, and partial oxidation of nitrogen functionalities. Photoluminescence decay profiles showed a modest increase in the average carrier lifetime, which may reflect changes in surface trap dynamics, possibly influenced by residual product adsorption. Importantly, the core structural integrity of mpg-CN remained intact, and the catalyst continued to function effectively across multiple cycles. Its compatibility with solvent recycling further underscores its suitability for sustainable oxidation processes. These findings highlight the robustness of mpg-CN and suggest that further studies on surface evolution could help optimize the long-term performance.

In addition, we compared different catalytic systems to compare the efficiency of our system with previous literature, including cumene and ethylbenzene as precursors (Supplementary Note 5). This clearly shows the superior conversion performances of our mpg-CN, even compared to systems with transition metal cocatalysts. Overall, these results highlight the potential of mpg-CN as a scalable, metal-free photocatalyst for aerobic oxidation reactions. While further optimization will be needed to address scale-up challenges toward large scale applications, this work provides a crucial foundation for developing industrially relevant, energy-efficient oxidation technologies with up to 6 orders of magnitude larger amount of precursor compared to conventional photocatalytic studies. Unlocking the full potential of this photocatalytic platform at scale will require targeted advances in reactor engineering, oxygen mass transfer, and catalyst design, critical levers for translating bench-scale efficiency into industrial viability. Unlike conventional methods that often rely on high temperatures, metal-based catalysts, or harsh oxidants, transition and rare metals cocatalysts, our approach operates under ambient conditions using molecular oxygen as the sole oxidating agent, along the lines of green chemistry principles. By validating the feasibility of larger-scale photocatalysis, this work paves the way for the development of environmentally friendly and energy-efficient oxidation processes, contributing to cleaner and more sustainable chemical manufacturing.

## 3 Conclusions

This study provides a comprehensive investigation of cumene oxidation via mpg-CN photocatalysis, focusing on reaction optimization, substrate scope, mechanistic elucidation, and catalyst stability. By systematically analyzing key reaction parameters, we establish fundamental design principles for enhancing selectivity, maximizing conversion, and enabling scalable photocatalytic oxidation under environmentally benign conditions. This work advances the field by identifying critical performance factors and proposing a mechanistic framework for the development of efficient, metal-free photocatalytic systems.

Substrate scope investigations revealed that the conversion efficiency is strongly influenced by radical stability, electronic effects, and oxygen availability. Toluene derivatives exhibited high selectivity, with electron-donating groups enhancing the benzylic radical stability and promoting efficient oxidation. In contrast, halogenated and heteroaromatic substrates showed reduced reactivity, likely due to unfavorable electronic effects and decreased radical lifetimes. Notably, ethylbenzene and 4-ethyltoluene maintained high conversion under ambient air conditions, unlike most substrates that required oxygen enrichment, indicating their enhanced susceptibility to benzylic oxidation and the formation of stable secondary radicals.

Mechanistic investigations, combining EPR spectroscopy with radical and peroxide scavenger assays, unequivocally demonstrate that the oxidation proceeds via a radical pathway initiated by photoinduced charge separation in mpg-CN. Upon visible-light excitation, mpg-CN generates electron–hole pairs; the photogenerated holes abstract hydrogen atoms from the benzylic position of cumene, forming cumyl radicals. These transient radicals rapidly react with molecular oxygen to produce hydroperoxyl species, which serve as key intermediates driving α-C–H oxidation. The essential role of these reactive oxygen species was confirmed through the complete suppression of product formation in the presence of radical and peroxide scavengers. Furthermore, EPR spectroscopy directly detected the formation of radical intermediates under operational conditions, providing compelling evidence for the proposed reaction pathway and reinforcing the role of mpg-CN as an efficient, metal-free platform for radical-based oxidations.

The scalability of mpg-CN photocatalysis was critically assessed, revealing key limitations that arise during reaction scale-up. Experiments identified photon flux attenuation, mass transfer constraints, and reduced radical formation efficiency as major barriers to maintaining high conversion at larger volumes. A 10-fold scale-up under standard conditions yielded 66% conversion and 70% selectivity after 24 h. However, further scale-up to 1 g of cumene in a custom-built batch photoreactor resulted in <10% conversion after 24 h, primarily due to reduced light penetration and oxygen diffusion. Extending the reaction time to 72 h increased the yield to 0.46 g of acetophenone (51%), highlighting the potential of the system while emphasizing the need for process optimization.

Collectively, these results not only establish mpg-CN as a promising platform for metal-free aerobic benzylic oxidation but also provide mechanistic and engineering principles critical for the advancement of next-generation photocatalytic systems. Unlike conventional oxidation methods that require elevated temperatures, transition metals, or harsh oxidants, this system operates under mild conditions, using molecular oxygen as the sole oxidant, aligning with the principles of green chemistry. We envision that it is further possible to expand the scope and tunability of the reaction by introducing an acid in the solution, expanding the scope of solvents, and increasing the reaction temperature, potentially changing the selectivity of the reaction or accessing different products, such as phenol. By validating the feasibility of photocatalytic oxidation at preparative scale, this work lays the foundation for the development of sustainable, energy-efficient processes for selective C–H bond functionalization toward industrial settings.

## 4 Materials and Methods

### Preparation of Catalyst

The process began by combining cyanamide (30 g) with a silica aqueous colloidal suspension (75 g, LUDOX HS-40, 40 wt %) until a homogeneous solution formed. This solution was then concentrated to a clear, amorphous solid through gradual evaporation with a rotary evaporator. Following this, the flask with the solid was quickly moved to a water bath, which was preset at 80 °C, and connected to a gas trap. During heating, the solid changed to white as ammonia was released. This white solid was then placed in a crucible and heated to 550 °C over 4 h in an oven, with a subsequent period of calcination at the same temperature for an additional 4 h under nitrogen flow. Once cooled, the solid was transferred to a polypropylene bottle containing NH_4_HF_2_ (120 g) and water (500 mL), stirred continuously for 24 h, and then filtered. The resulting solid was thoroughly rinsed with water and ethanol and left to dry overnight in a vacuum oven. The total yield of the process was 16.0 g.

### General Catalytic Tests

A 5 mL vial was fitted with a magnetic stirrer and filled with mpg-CN (20 mg) and 3 mL of acetonitrile. Subsequently, cumene (0.005 mmol) was added, and all was mixed thoroughly. The vials were sealed with screw caps equipped with a PTFE liner and purged with O_2_ using a double needle technique. The reaction mixture was then irradiated for 24 h inside a blue photoreactor (λ_EM_ = 456 nm, *E*_EM_ = 80 mW cm^–2^). After the reaction, the catalyst was separated by centrifugation, and the solution was transferred back into the vial for analysis by GC-MS.

For the experiments with different substrates, 0.005 mmol of tested compound was added to a vial filled with mpg-CN (20 mg) and 3 mL of acetonitrile.

For the experiments performed in ambient air, filled vials were sealed with crew cups without additional purging.

### General Procedure for Catalyst Stability Tests

The reaction was performed in a 5 mL vial as previously described. After 24 h of irradiation, the catalyst was recovered by centrifugation, washed with ethanol, and dried at 60 °C in a vacuum oven. After each catalytic run, the reaction mixture was collected and analyzed by GC-MS.

### General Procedure for Solvent Recirculation

The reaction was carried out in a 15 mL glass reactor containing 0.250 mmol of cumene and 100 mg of catalyst. Prior to irradiation, the reaction mixture was purged with oxygen for approximately 5 min using the double-needle technique. After 24 h of irradiation, the solvent was removed via rotary evaporation and reused in the subsequent catalytic cycle, where a fresh batch of catalyst and cumene was introduced into the reactor. Following each catalytic run, the reaction mixture was collected and analyzed by GC-MS.

### General Procedure for Scale-Up Experiment

A custom-built photoreactor was used for the scale-up experiment, designed to accommodate larger reaction volumes while ensuring efficient light exposure and oxygen diffusion. The reactor was equipped with a 40 L oxygen balloon to maintain a continuous oxygen supply, and a magnetic stirrer was used to ensure proper mixing and mass transfer. In the photoreactor, 1 g of cumene (8.4 mmol) was dissolved in 200 mL of acetonitrile under ambient conditions. 800 mg of mpg-CN photocatalyst was added, and the mixture was stirred continuously. The system was irradiated using three custom-made LED lights (λ_EM_ = 456 nm, *E*_EM_ = 80 mW cm^–2^) placed in distance approximately 5 cm from the reactor, while oxygen was continuously supplied from the 40 L balloon, which was refilled every 24 h. After 24 h, a small aliquot was taken for GC analysis to monitor acetophenone formation. Due to low conversion, the reaction was extended to 72 h under the same conditions, with regular oxygen replenishment. Upon completion, the reaction mixture was filtered to remove the solid catalyst.

### Isolation and Purification of the Product

The filtrate was concentrated under reduced pressure to remove the solvent. The resulting crude product was purified by column chromatography using dichloromethane as the eluent. Acetophenone (0.46 g, isolated yield) was obtained as the final product.

### Material Characterization

The crystallinity of the samples was analyzed using a Rigaku SmartLab powder X-ray diffraction (XRD) system (Japan, Cu Kα, 0.154 nm). Fourier-transform infrared (FTIR) spectra were obtained with a Thermo Scientific Nicolet iD7 (USA) spectrometer. Fluorescence spectra were recorded by using a Jasco FP-830 instrument (Japan) with an excitation wavelength of 375 nm. Optical absorbance of the powders was measured on a Shimadzu UV 2600 instrument equipped with an integrating sphere. Electron Paramagnetic Resonance (EPR) studies were carried out on a Bruker EMXnano benchtop X-Band EPR spectrometer (Germany) using the following settings: Center Field 3448.05 G, Sweep Width 200 G, Receiver Gain 50 dB, Modulation Amplitude 1.000 G, Number of Scans 10, and Microwave Attenuation 20 dB. Samples were placed in flame-sealed EPR capillaries (IntraMark, volume 50 μL, ID 0.86 mm) within an EPR tube (ID 3 mm, OD 4 mm, length 250 mm). Obtained spectrum was fitted using SpinFit software (Brukerm Germany) containing a spin-trapping library of common spectra. Scanning Electron Microscopy (SEM) imaging was performed using the Zeiss LEO 1550-Gemini (Germany) system with an acceleration voltage of 10 kV. Mass spectral data were obtained from an Agilent GC 8890 gas chromatograph coupled with an Agilent GC/MSD 5977B mass spectrometer (electron ionization), using an HP-5MS column (inner diameter = 0.25 mm, length = 30 m, and film = 0.25 μm). Gas analysis was conducted using Gas Chromatography coupled with a Thermal Conductivity Detector (GC-TCD) of the type QP2010 by Shimadzu using GC Real Time Analysis for recording and LabSolutions by Shimadzu for analysis. The measurement parameters were used as stated below. column: Carboxen-1010 PLOT, 30 m x 0.53 mm I.D., oven parameters: 35 °C (7.5 min) to 250 °C at 24 °C/min, injection temperature: 200 °C, detector: TCD, 230 °C; flow rate: Argon, 3.0 mL/min. To investigate the chemical composition of the nanoparticle surface, X-ray photoelectron spectroscopy (XPS, XPS UHV system from PREVAC Sp. z o. o., Al-anode, 300W, no monochromator, main Alkalpha energy = 1486.6 eV) was utilized. Survey scans were conducted at 3 iterations and a pass energy of 200 eV while high-resolution scans were performed at 20 iterations and a pass energy of 50 eV. For the analysis of XPS spectra, the software CasaXPS (version 2.3.23) was used. Charge correction was performed by referencing all spectra to the C 1s peak of adventitious carbon at 285.0 eV.

## Supplementary Material



## Notes

Methods; material characterization; photocatalytic and reusability studies; products characterization; comparison with other photocatalyst; additional references (PDF)
