# Supplementary material for: One-Step Photocatalytic Oxidation of Cumene with Mesoporous Carbon Nitride
Source: ACS Catal. 2026 Mar 2;16(6):5654–63. doi: 10.1021/acscatal.5c08511 (PMC13011261; doi:10.1021/acscatal.5c08511)
Supplement: Supplementary file 1 [file cs5c08511_si_001.pdf]

Supplementary Information

One-step Photocatalytic Oxidation of Cumene with Mesoporous Carbon Nitride

Sonia Żółtowska[1], Gabriel Chan[1], Tim Tjardts[2], Paolo Giusto*[1,3]

[1] Department of Colloid Chemistry, Max Planck Institute of Colloids and Interfaces, 14476 Potsdam, Germany

[2] Institute of Material Science, Christian-Albrechts-Universität zu Kiel, 24143 Kiel, Germany

[3] Queensland University of Technology, School of Chemistry and Physics, Joint Laboratory on Nanocatalysis for Sustainable Chemistry, Brisbane, Queensland 4000, Australia

Email: paolo.giusto@mpikg.mpg.de

Table of Contents

Scale up reactions for ethyl acetate S2

Reusability study and comparative analysis of spent catalyst S3

Surface area characterization of fresh catalyst S8

Comparison with other catalytic systems S10

Spectroscopic data of chemicals S13

Supplementary note 1: Scale-up experiments for ethyl acetate

General procedure

A 200 mL three-neck glass reactor, equipped with a magnetic stirring bar, was loaded with mpg-CN (750 mg), cumene (650 mg) and ethyl acetate (150 mL). O_2-enriched atmosphere was provided by connecting a O_2-filled balloon to the reactor, creating a constant flow of O_2 of 1 bar. The reaction mixture was then irradiated using 2× 465 nm blue LED, one on each flat side of the reactor, for 120 h with active fan cooling. An aliquot was taken after the first 24 h to confirm catalytic activity, and the reaction was allowed to proceed.

After completion of reaction, the reaction mixture was transferred to centrifuge tubes and centrifuged for 10 mins to separate the catalyst. The organic parts were combined and placed into a 250 mL round-bottom flask, where a portion of 20 μL was taken as GC-MS sample, and the rest being concentrated with a rotary evaporator.

For the GC-MS analysis, 20 μL of the reaction mixture was diluted 10 times. An internal standard of 0.9 μL octadecane was added into the GC-MS sample, and the mixture was injected into the GC-MS system.

Supplementary note 2: Reusability study and comparative analysis of spent catalyst

To assess the long-term stability and reusability of the mpg-CN photocatalyst, a multi-cycle oxidation study was conducted. The catalyst was used in three consecutive cycles of cumene photooxidation, both in ambient air and under an oxygen-enriched atmosphere (Fig. S1). Evaluating the catalytic performance of the catalyst over multiple cycles provides insights into its durability, deactivation mechanisms, and potential for practical applications.

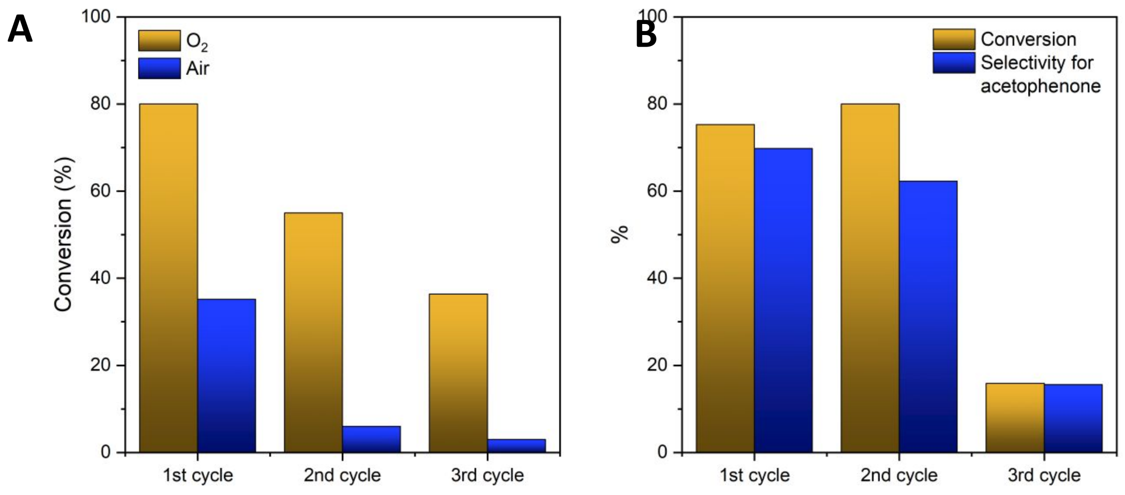

Fig. S1. Reusability study of the catalyst with oxygen (O_2) and air (A); reusability study of acetonitrile (B).

As expected, in all three cycles (Fig. S1A), cumene photooxidation achieves significantly higher conversion under pure oxygen than in air. However, a gradual decline in conversion is observed across successive cycles, indicating progressive catalyst deactivation. This decrease may be attributed to several factors, including surface oxidation, and loss of active sites due to structural degradation. These changes reduce the catalyst's ability to generate ROS, thereby hindering oxidation efficiency (see Fig. S2-S3). The significantly lower conversion in air suggests that oxygen concentration directly influences the rate of ROS formation, which is essential for cumene oxidation. In an oxygen-enriched atmosphere, a higher partial pressure of O_2 facilitates the generation of superoxide radicals ($O_2^{\bullet -}$) and hydroperoxyl radicals ($^{\bullet}OOH$), which are key oxidative intermediates. In contrast, under ambient air conditions (~21% O_2), the reduced availability of oxygen limits ROS formation, thereby lowering overall conversion efficiency.

In line with the sustainability principles of photocatalysis, assessing solvent reusability was a critical aspect of this study. The ability to recover and reuse the solvent across multiple catalytic cycles is essential for improving the economic and environmental viability of the process. To evaluate this, we scaled up the reaction fourfold and, after each run, recovered acetonitrile through simple vacuum evaporation, reusing it in subsequent photocatalytic cycles. As shown in Figure S1B, a significant decrease in conversion was observed after the third reuse of acetonitrile, indicating a gradual decline in solvent efficiency. This reduction could be attributed to the accumulation of by-products, reducing the solubility of oxygen, affecting the overall performance of the photocatalytic system. Despite this, our results highlight that the

proposed mpg-CN-based catalytic system demonstrates potential for sustainable photooxidation processes, with solvent recycling offering a feasible route to minimize waste and enhance process efficiency.

To gain deeper insight into the mechanism of catalyst deactivation, it is essential to examine the structural and physicochemical changes occurring after multiple reaction cycles. Figure S2 presents a comparative analysis of the optical properties using UV-Vis spectroscopy (UV-Vis) and photoluminescence spectroscopy (PL), the bulk structural characteristics via X-ray diffraction (XRD), and the surface composition and functional groups examined through X-ray photoelectron spectroscopy (XPS) and Fourier transform infrared spectroscopy (FTIR) for both fresh and spent catalysts.

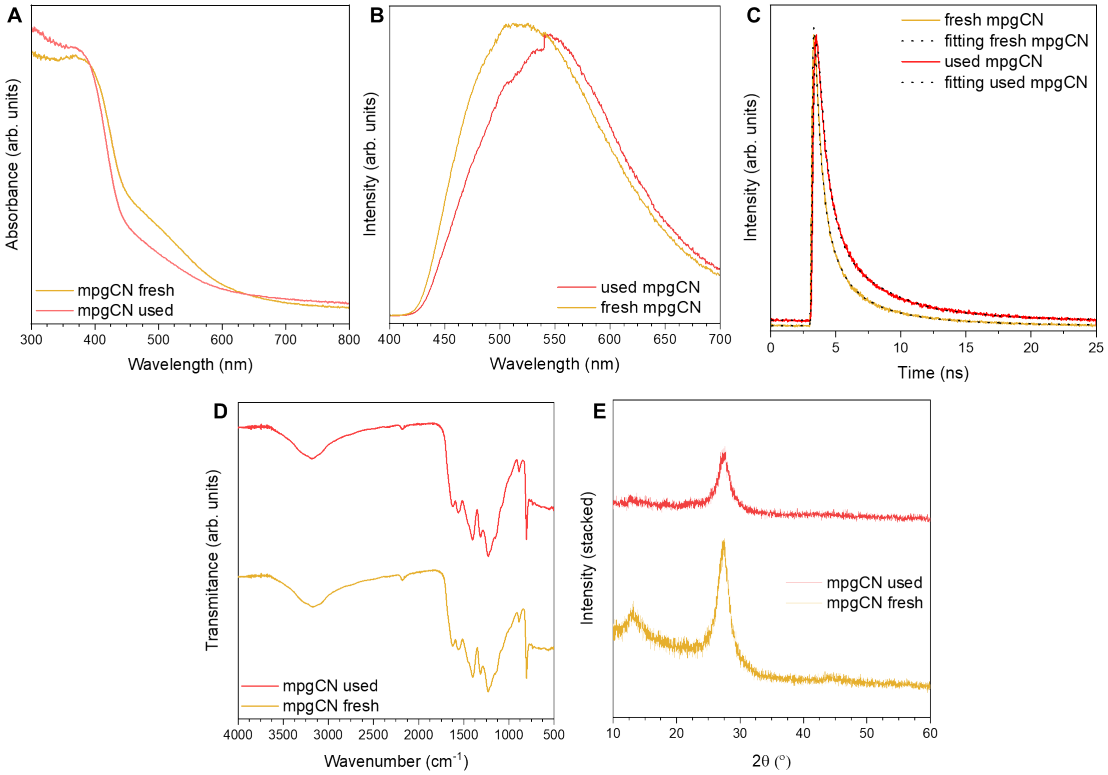

Fig. S2. Comparison of spent catalyst with freshly prepared mpg-CN used in this study.

The optical properties of mpg-CN also undergo noticeable changes after catalytic use, as shown in Fig. S2A-B. The used mpg-CN (red spectrum) exhibits a minor shift in the absorption edge, which can be attributed to surface oxidation and defect formation. Oxygen incorporation into the mpg-CN framework introduces localized electronic states, altering charge carrier dynamics [1]. Additionally, the formation of oxidized species disrupts the extended conjugation of the π-electron system, which reduces electronic delocalization and leads to a slight increase in band gap energy [1]. Surface oxygen species may also act as surface trap reducing electron transfer efficiency to the substrate. Furthermore, the increased absorbance in the UV region for the used catalyst provides additional evidence of surface oxidation. The shift towards higher wavelengths on the PL spectrum is a strong indication of surface oxidation [2, 3]. This can be related to an increase in non-radiative recombination sites, where trapped electrons and holes lead to a longer emission wavelength. Such changes imply that some degree of catalyst aging

and deactivation may occur over successive cycles. Figure S2C shows the radiative lifetimes of photogenerated charge carriers in mpg-CN before and after catalytic reactions. We analyzed the fluorescence decay profiles using a biexponential fitting model. For the fresh catalyst, we observed two characteristic lifetimes: a short-lived component (τ_1=0.2 ns) and a longer-lived component (τ_2=13.36 ns), resulting in an average lifetime (τ_{avg}) of 3.1 ns. In contrast, the spent catalyst exhibited τ_1=0.68 ns and τ_2= 4.68 ns, with a corresponding τ_{avg} of 3.8 ns. The rapid initial decay (τ_1) is generally attributed to recombination processes occurring near or at the surface of inorganic semiconductors, while the extended τ_2 reflects delayed recombination dynamics, likely arising from trap states within the band gap that prolong charge carrier lifetimes. The increased PL lifetime of the spent catalyst likely arises from partial adsorption of reaction products, which alters the local surface environment by affecting the density and distribution of trap states.

The FTIR spectra of fresh (brown) and used (red) mpg-CN reveal only minor differences related to increasing intensity of O–H stretching bands (3200–3500 cm^{-1}). This suggests the formation of hydroxylated species, possibly due to surface oxidation. These findings uphold the hypothesis that oxidative surface modifications contribute to catalyst deactivation [4, 5].

The comparison between the fresh and spent catalyst reveals structural and chemical transformations after catalytic use. XRD analysis shows significant reduction of the 12° peak in the spent catalyst, which corresponds to the (100) plane, associated with the in-plane structural order of tri-s-triazine units. Such change might suggest changes of in-plane ordering within the carbon nitride framework, likely due to partial structural degradation caused by prolonged exposure to reactive oxygen species (ROS) [6-8]. However, the 27° peak remains, suggesting that while the in-plane order is disrupted, the overall layered structure of mpg-CN is preserved, although with possible modifications. These observations indicate that catalyst deactivation primarily results from structural disordering rather than complete framework collapse.

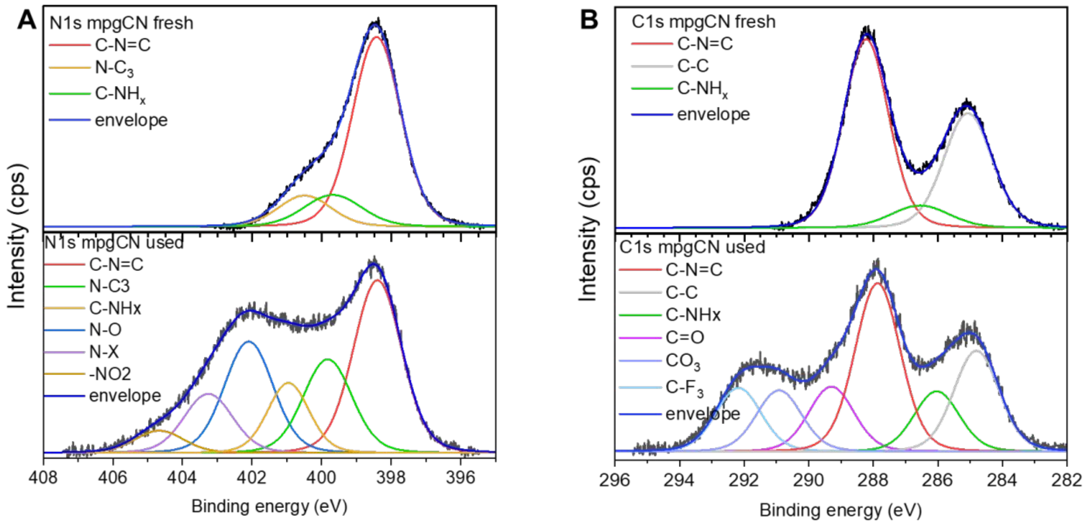

Fig. S3. N1s (A) and C1s (B) XPS spectra of fresh (upper part) and spent mpgCN bottom part).

Further insights into the surface chemistry evolution of mpg-CN after photocatalytic reactions are provided by XPS analysis (Fig. S3). The C 1s and N 1s core-level spectra of the fresh catalyst exhibit well-defined peaks characteristic of carbon nitride, consistent with its well-ordered tri-s-triazine structure [9]. However, after catalytic use, modifications in chemical states and electronic structure are observed. In the N 1s spectrum of the used catalyst, peak broadening and an increase in the population of C–NHx groups suggest a higher concentration of free CN groups, overlapping with contributions from C–NHx species. Additionally, the shift toward higher binding energies points to an overall oxidation of the system making it more electron depleted, likely induced by surface oxidation. New features associated with N-O, and -NO_2 species are also detected [10], indicating partial surface oxidation of the nitrogen sites within the mpg-CN framework. In addition, N-X oxidized species have been found assigned to trapped, charged N that might also contain contributions from the π-π* shake-up satellite feature.

These observations are further supported by the C 1s spectrum of the used catalyst, which shows the appearance of new peaks corresponding to C=O and CO_3 species, confirming the formation of oxygen-containing functional groups on the surface. In addition, traces of fluorine were observed, likely originating from the removal of the template during the synthesis process. In presented case, C 1s and N 1s core levels remain characteristic of mpg-CN. Moreover, the population of C-NH_x species increases, indicating a change in the ratio of C-NHx to CN groups [10, 11].

Morphological changes in mpg-CN were analyzed using scanning electron microscopy (SEM). As shown in Figure S4, after three catalytic cycles, noticeable alterations in the catalyst's morphology were observed. Significant particle agglomeration has occurred, resulting in the formation of larger, denser clusters. Additionally, the catalyst surface appears rougher and less uniform, indicating structural degradation after repeated use. Furthermore, the originally well-defined mesoporous structure shows signs of collapse in several regions, with the surface becoming more compact. These morphological changes are consistent with the catalyst deactivation mechanisms discussed earlier, including aggregation, pore blockage, and loss of active surface area.

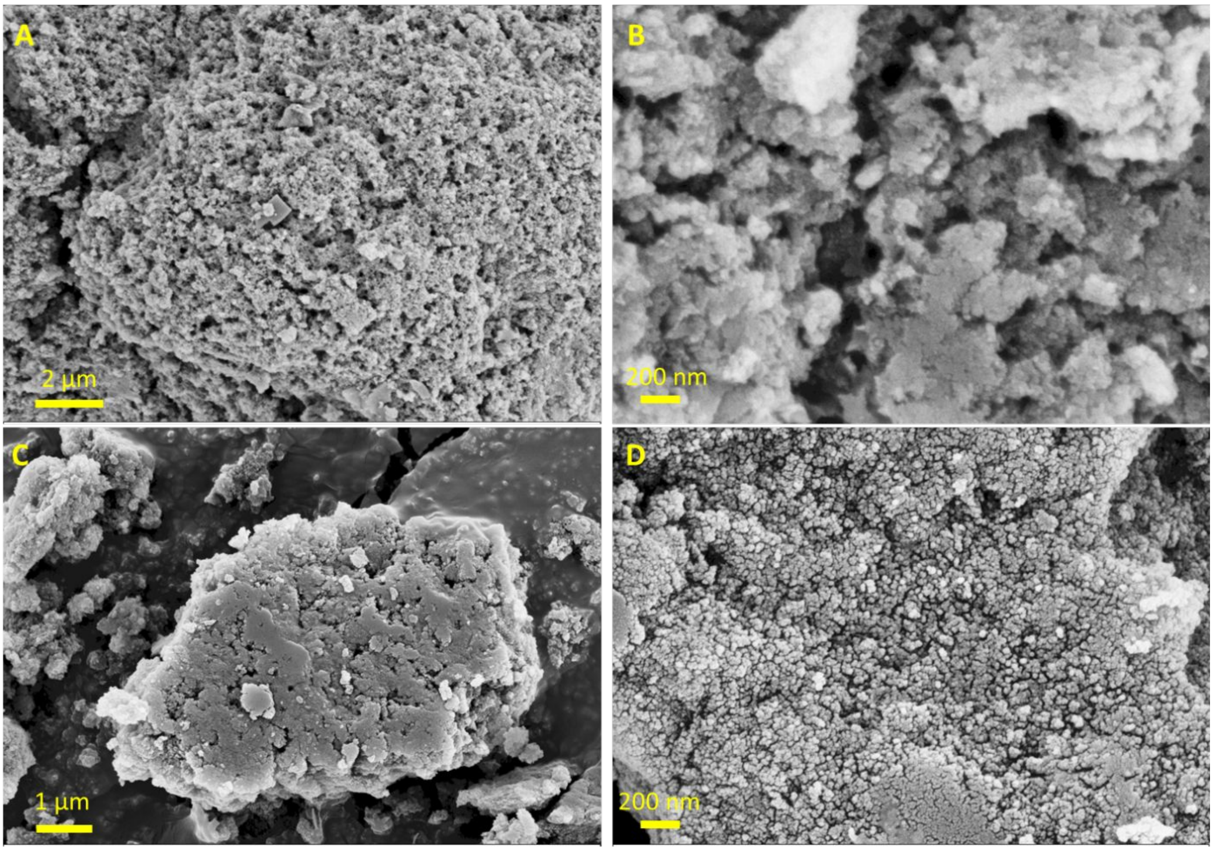

Fig. S4. SEM images of fresh (A, B) and spent catalyst (C, D). Pictures a different magnification.

The combined XRD, UV-Vis, FTIR, XPS and SEM analyses provide clear evidence that photocatalytic use leads to structural disordering, surface oxidation, and electronic structure modifications in mpg-CN. These changes impact the catalyst's stability and efficiency, ultimately contributing to deactivation over multiple reaction cycles.

Table S1. Solubility and dielectric constant values of solvents used in photocatalytic cumene oxidation.[12]

**Solvent**	c_{O2}**, mM**	**Dielectric constant**
Acetonitrile	2.60	42.30
Ethyl acetate	2.06	6.02

https://pubs.rsc.org/en/content/articlelanding/2013/an/c3an36782g#cit12The

The oxygen solubility in air-saturated solvents at room temperature (0.21 atm O_2) was approximately 2.06 mM in ethyl acetate and 2.6 mM in acetonitrile, consistent with the higher polarity and oxygen solubility of acetonitrile.

Supplementary note 3: Surface area characterization of fresh catalyst

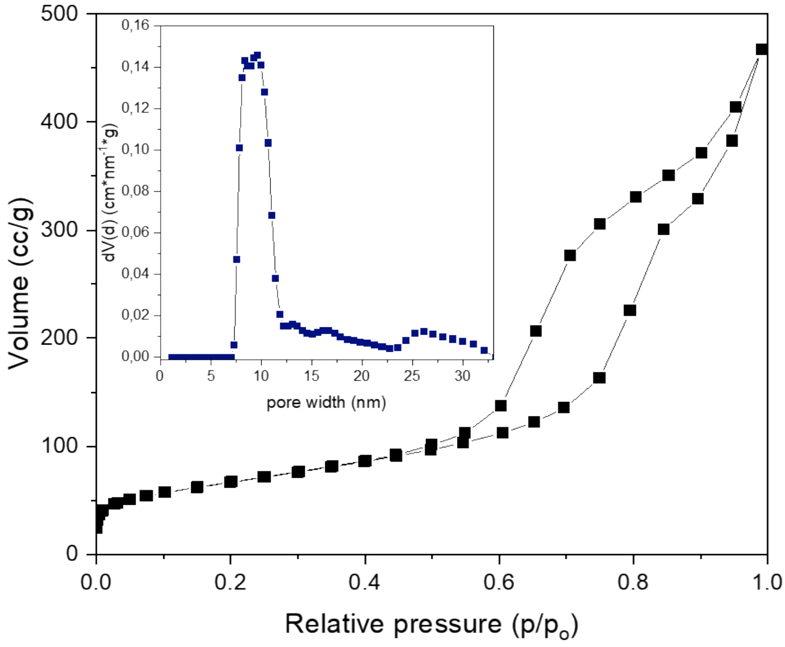

Fig. S5. N_2 adsorption-desorption isotherm.

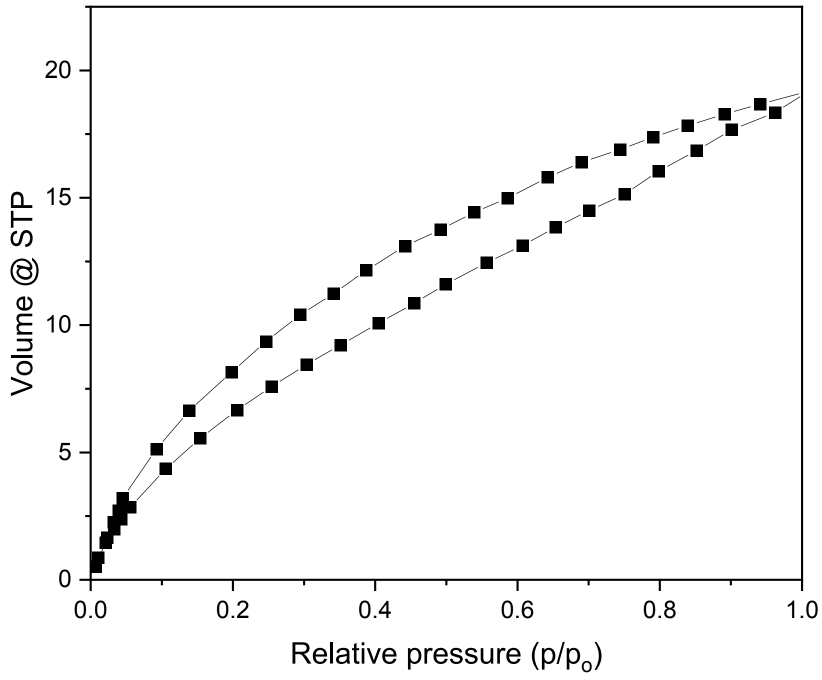

Fig. S6. CO_2 adsorption-desorption isotherm.

Specific surface areas (SSA) were determined from N2 adsorption-desorption isotherms using the Brunauer–Emmett–Teller (BET) method (Fig. S5). The synthesized mpg-CN exhibited a high surface area of 260 m2/g, characterized by a Type IV isotherm with an H2-type hysteresis loop, typical of mesoporous architectures with complex pore connectivity. Pore size distributions, calculated via the Barrett–Joyner–Halenda (BJH) method, revealed a primary mesopore population ranging from 6 to 15 nm. The significant broadening of the BJH peak, corroborated by CO2 sorption analysis, indicates a contribution of micropores. Furthermore, a secondary distribution of larger pores was observed in the 25-30 nm range.

The expansion of surface area is a fundamental strategy to mitigate the inherent limitations of bulk graphitic carbon nitride, which is frequently constrained by restricted active sites and rapid charge carrier recombination.[13] For covalent carbon nitrides, the increasing of the porosity enhances the photocatalytic activity by shortening the diffusion distance of photogenerated carriers to reach the surface, thereby reducing recombination effects. [14, 15]

Supplementary note 5: Comparison of other catalytic systems

Table S2. Comparison of different photocatalytic systems for oxidation of cumene and ethylbenzene

Catalyst	Solvent	Conditions	Oxidant	Additional reagent(s)	Wavelength	Starting reagent	Conversion (%)	Selectivity (%)	Yield (%)	Reference
mpg-CN	Acetonitrile	substrate 0.05 mmol, catalyst 20 mg, room temperature, 24 h	O_2	--	Blue (456 nm)	Ethylbenzene	95	99	94	This work
mpg-CN	Acetonitrile	substrate 0.1 mmol, mpg-CN (6 mg), 25–30 °C, 16 h	O_2	1 equiv of Na_2CO_3	Blue (456 nm)	Ethylbenzene			68	[16]
CN-OA-m	Potassium sulphate buffer	substrate 10 mmol, CN-OA-m 2 mg/mL, 30 °C, 8 h	O_2	MeOH (1% v/v)	Blue (455 nm)	Ethylbenzene	(product not isolated)			[17]
$CsPbBr_3$/Au/g-C_3N_4	1,2-dichloroethane	substrate 0.2 mmol, $CsPbBr_3$/30Au/CN (15 mg), 10 h	O_2	--		Ethylbenzene	82	89		[18]
$BiVO_4$/Ag/g-C_3N_4	Acetonitrile	substrate 1 mmol, photocatalyst 50 mg, room temperature, 3 h	O_2	--	Visible light	Ethylbenzene		99	150 (μmol/g·h)	[19]

g-C_3N_4	Acetonitrile	substrate 1 mmol, g-C_3N_4 100 mg, 60 30 °C, 23 h	O_2	NHPI (0.1 equiv.)	Visible light	Ethylbenzene	27	84		[20]
VO/g-C_3N_4	Acetonitrile	substrate 1 mmol, VO/g-C_3N_4 25 mg, room temperature, 12 h	H_2O_2	--	Domestic bulb (40 W)	Ethylbenzene			99	[21]
g-C_3N_4	Acetonitrile	substrate 0.5 mmol, g-C_3N_4 5 mg, room temperature, 6 h	O_2	--	Blue (455 nm)	Ethylbenzene			96	[22]
V NPs/g-C_3N_4	H_2O	substrate 0.1 mmol, V NPs/g-$C_3N_4$5 mg, room temperature, 12 h	TBHP	--	Domestic light (50 W)	Ethylbenzene	99	99		[23]
Benzylamine-decorated g-C_3N_4	Acetonitrile	substrate 0.1 mmol, catalyst 10 mg, room temperature, 8 h	O_2	--	Purple (420 nm)	Ethylbenzene	95	93		[24]
$Fe^{III}(Q_b)_3$/g-$C_3N_4BF_{0.5}$	Acetonitrile	substrate 1 mmol, catalyst 0.01 mmol, additive 10	H_2O_2	--	Visible light (35 W)	Ethylbenzene	13		9	[25]

		mg, 18-20 °C, 12 h								
Co/g-C_3N_4	Acetonitrile/ H_2O (1:1)	substrate 0.1 mmol, catalyst 5 mg, 60 °C, 15 h	O_2	PMS (5 equiv.)		Ethylbenzene	98	96		[26]
Br-bridged g-C_3N_4	Acetonitrile	substrate 20 mmol, catalyst 0.18 g, room temperature, 24 h	O_2	CHP (0.15 equiv.) K_2CO_3 (0.05 equiv.)	Xenon lamp (300 W)	Ethylbenzene	43	92		[27]
$Sc(OTf)_3$ with riboflavin tetraacetate	Acetonitrile	substrate 0.02 mmol, catalyst 4.6 mM, room temperature, 2.5 h	Air	37% HCl	Blue (440 nm)	Ethylbenzene	100	60		[28]
Sandwich-like g-C_3N_4	--	substrate 20 mmol, initiator 2 mmol, catalyst 0.18 g	O_2	Cumene hydroperoxid e	Blue	Cumene	26	3		[29]
g-C_3N_4	DMSO-d6	substrate 0.2 mmol, catalyst 20 mg, room temperature, 24 h	O_2	--	Violet	Cumene			28	[30]
PAN/Ag NPs/g-C_3N_4 Nanofibres	Acetonitrile	substrate 1 mmol, catalyst 20 mg, 24 h	H_2O_2	--	Domestic bulb (40 W)	Cumene	98	99		[31]

Co/g-C_3N_4	--	substrate 20 mmol, catalyst 0.18 g, 20 h	O_2	K_2CO_3 (0.05 equiv.)	Blue (450 nm)	Cumene	52	8		[32]

Supplementary note 6: Spectroscopic data of chemicals

Cumene (isopropylbenzene) [33]

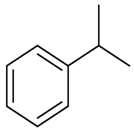

From reference: ^{1}H NMR (250 MHz, $CDCl_3$) δ 7.38 – 7.02 (m, 5H); 3.03 (septet, 1H); 1.40 (d, 6H)

Our measurement: ^{1}H NMR (400 MHz, $CDCl_3$) δ 7.46 – 7.30 (m, 5H); 3.06 (septet, 1H); 1.40 (d, 6H)

Acetophenone [34]

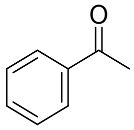

From reference: ^{1}H NMR (400 MHz, $CDCl_3$) δ 7.97 (d, 2H); 7.57 (t, 1H); 7.47 (t, 2H); 2.62 (s, 3H)

Our measurement: ^{1}H NMR (400 MHz, $CDCl_3$) δ 8.13 (m, 2H); 7.77 (tt, 1H); 7.65 (m, 2H); 2.73 (s, 3H)

Supplementary note 7: NMR Spectra of scale-up reaction in MeCN

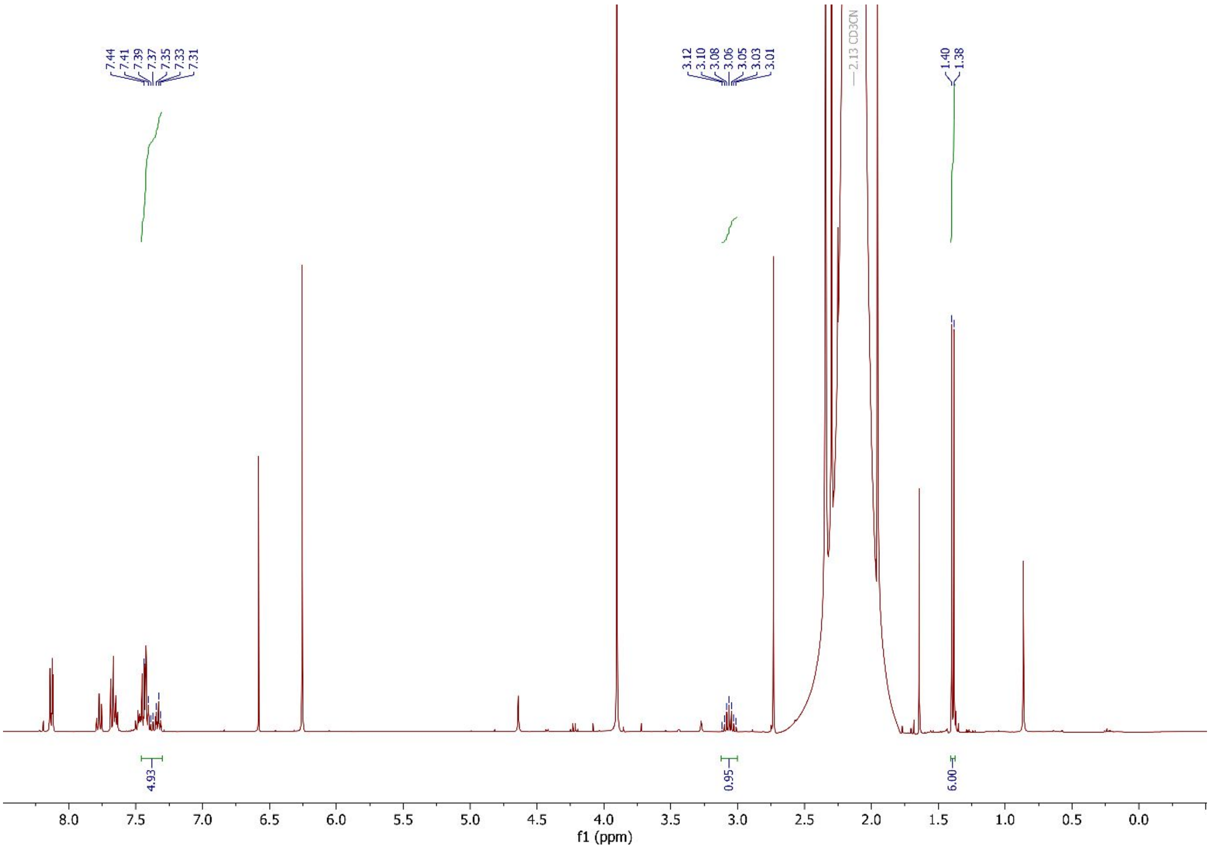

^{1}H NMR Spectrum of scale-up reaction performed in acetonitrile, highlighting the characteristic peaks of cumene.

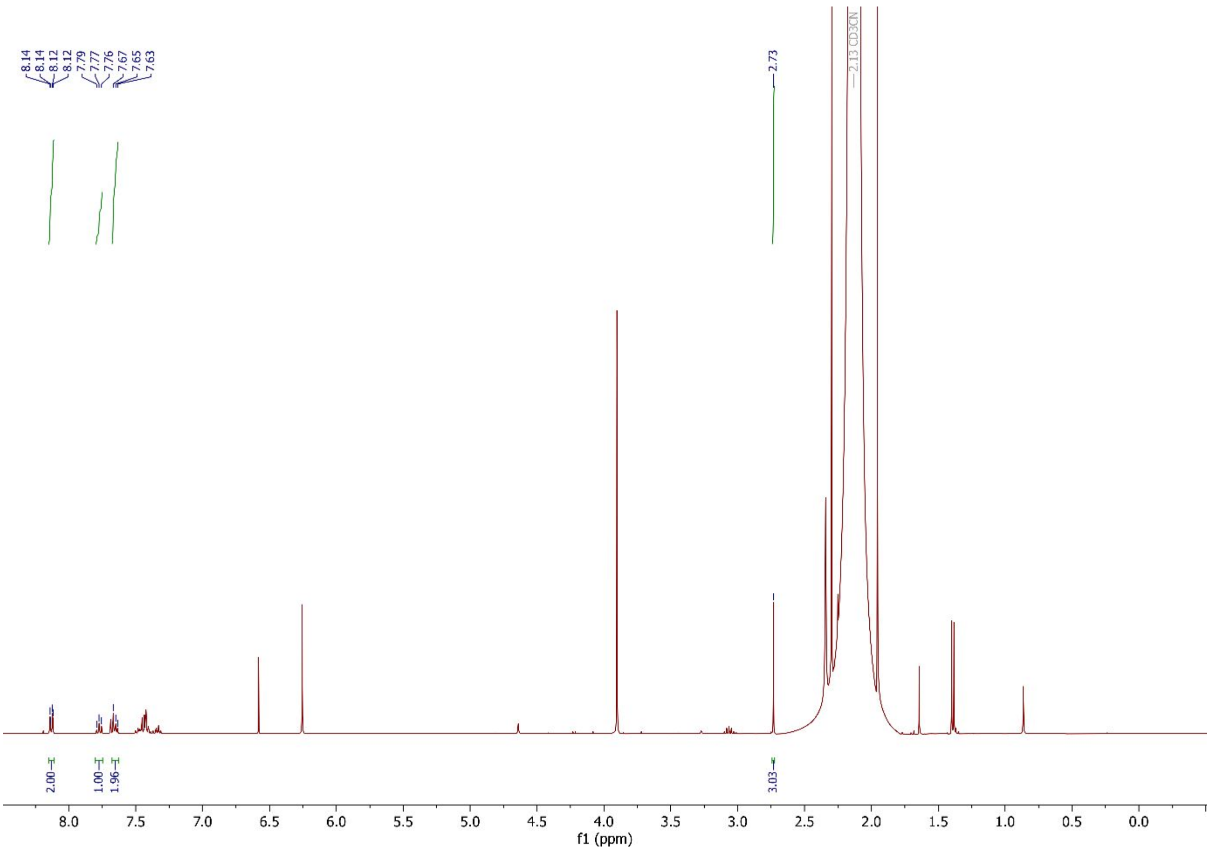

^{1}H NMR Spectrum of scale-up reaction performed in acetonitrile, highlighting the characteristic peaks of acetophenone.

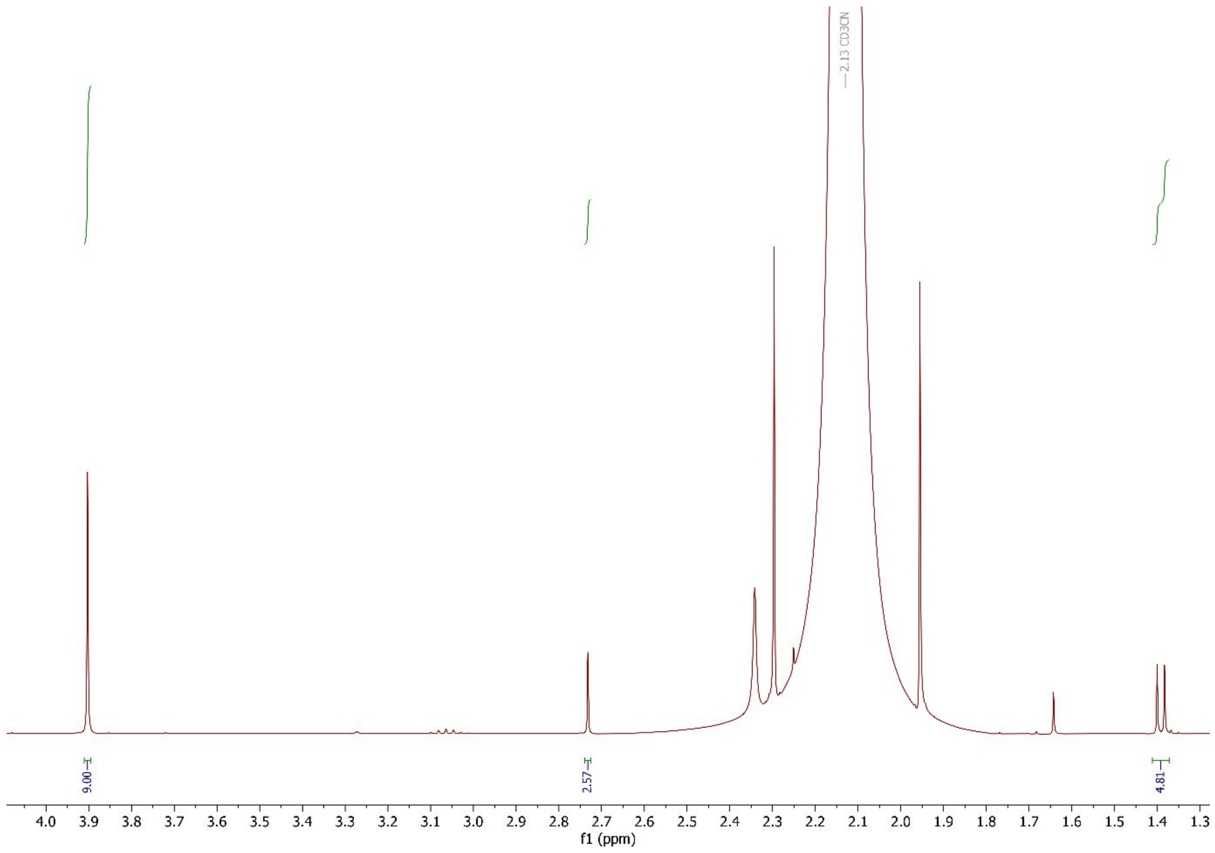

^{1}H NMR Spectrum of scale-up reaction performed in acetonitrile, with integration used to calculate yield and conversion.

A characteristic peak is chosen from cumene (δ 1.40, d, 6H) and acetophenone (δ 2.73, s, 3H). Internal standard was 1,3,5-trimethoxybenzene (δ 3.90, s, 9H) in 60 mol% of the initial cumene concentration (5 mg/mL = 0.0416 mmol mL^{-1}).

Yield was calculated as the following:

$$Yield = \frac{Integrated\ \#\ of\ H\ from\ characteristic\ peak}{\#\ of\ H\ represented\ by\ the\ characteristic\ peak} \times mol\%\ of\ internal\ standard$$

Which, in the case of acetophenone, is:

$$Yield = \frac{2.57}{3} \times 60\% = 51.4\ \%$$

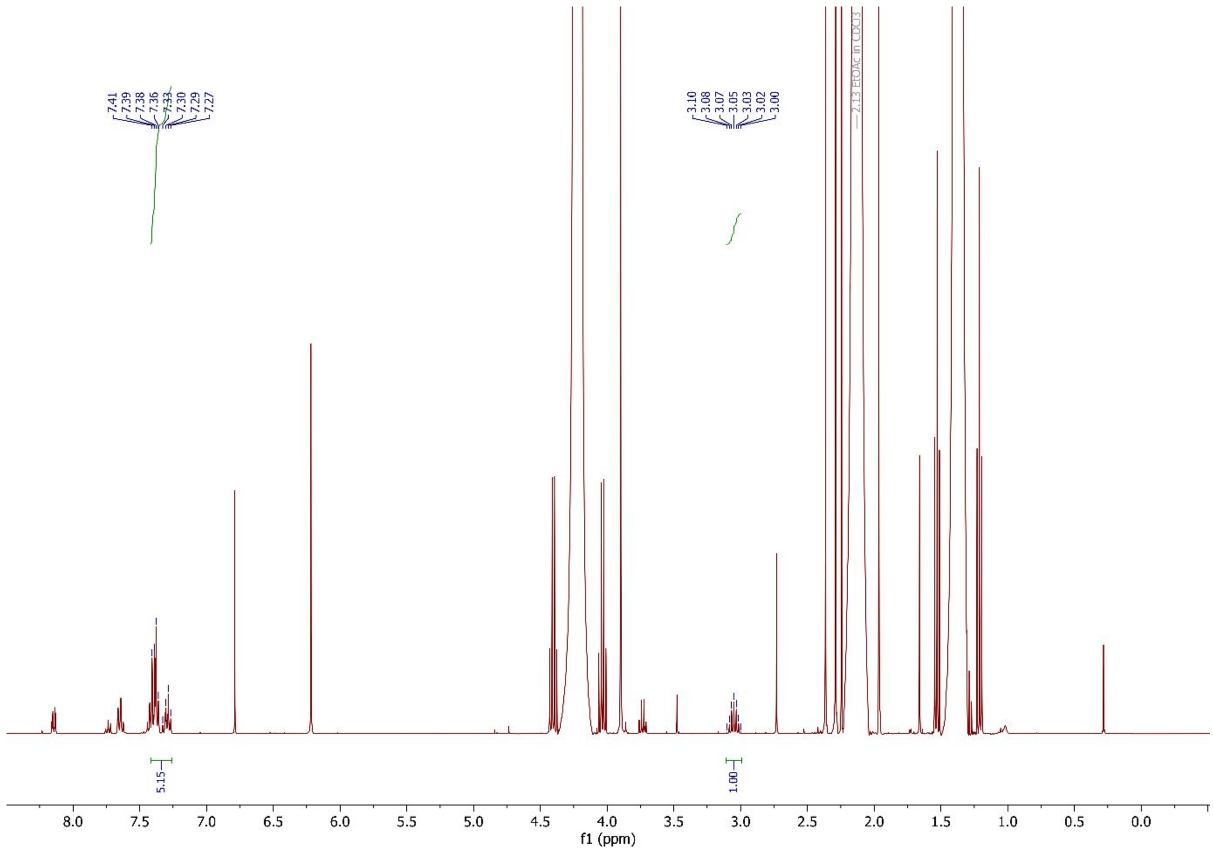

^{1}H NMR Spectrum of scale-up reaction performed in ethyl acetate, highlighting the characteristic peaks of cumene. The peak of ca. δ 1.40 (d, 6H) is overshadowed by residual ethyl acetate peak in the same area and could not be highlighted.

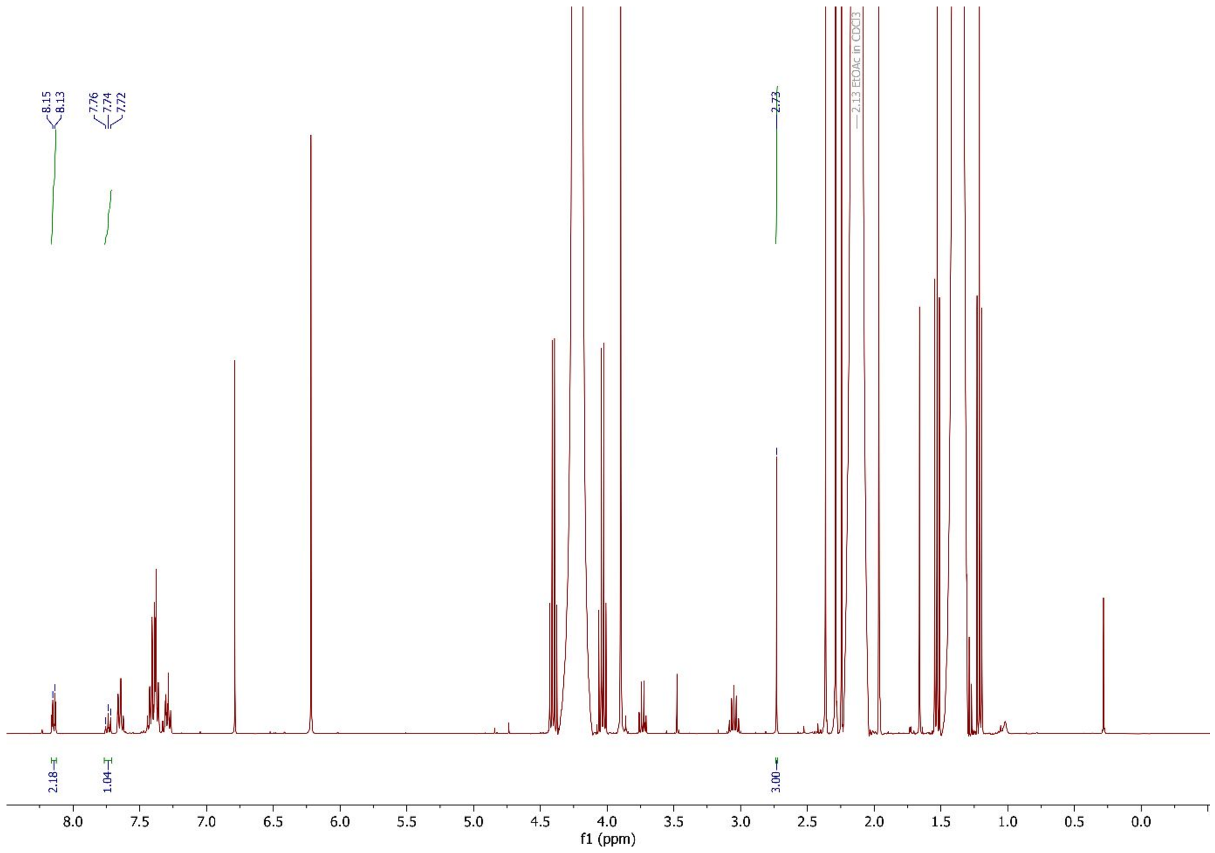

^{1}H NMR Spectrum of scale-up reaction performed in ethyl acetate, highlighting the characteristic peaks of acetophenone.

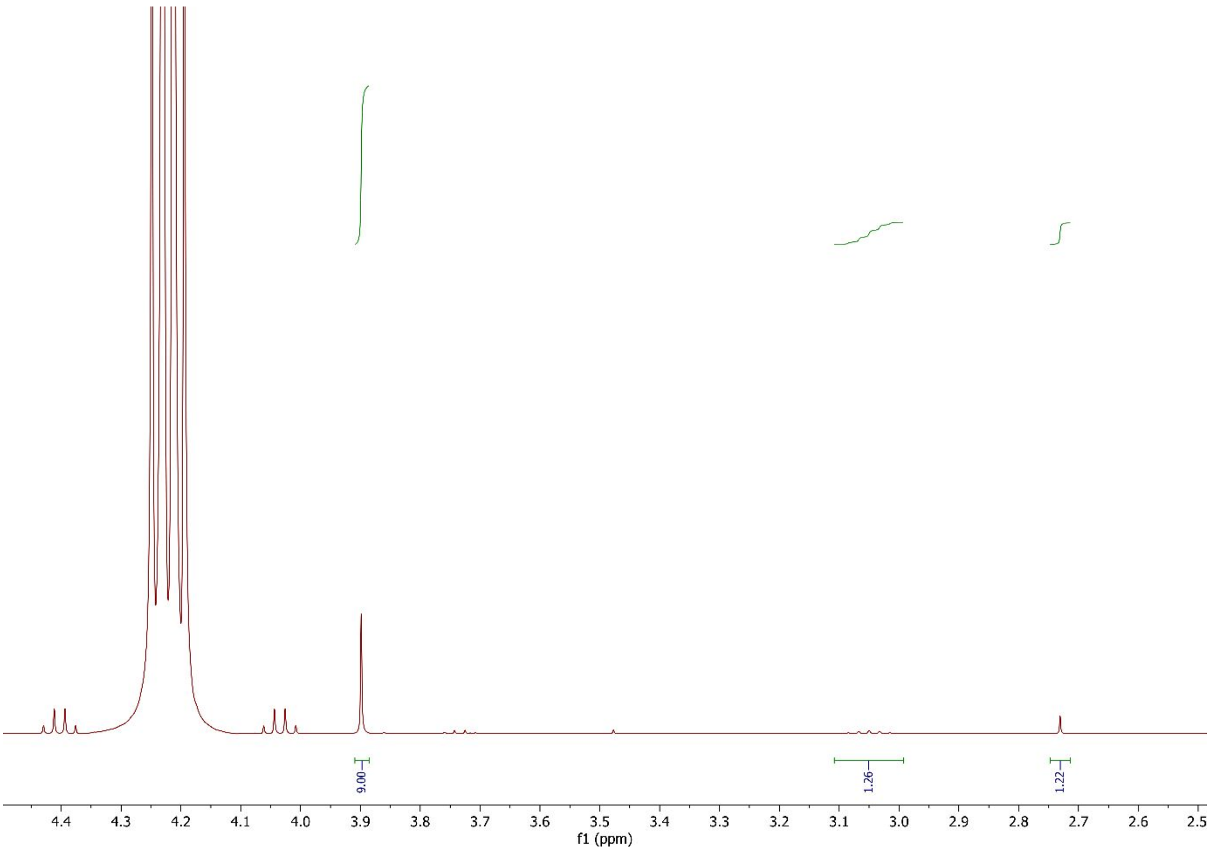

^{1}H NMR Spectrum of scale-up reaction performed in ethyl acetate, with integration used to calculate yield and conversion.

A characteristic peak is chosen from cumene (δ 2.96, septet, 1H) and acetophenone (δ 2.65, s, 3H). Internal standard was 1,3,5-trimethoxybenzene (δ 3.81, s, 9H) in 68.5 mol% of the initial cumene concentration (4.33 mg/mL = 0.0361 mmol mL^{-1}).

Yield was calculated as the following:

$$Yield = \frac{Integration\ \#\ of\ H\ from\ characteristic\ peak}{\#\ of\ H\ represented\ by\ the\ characteristic\ peak} \times mol\%\ of\ internal\ standard$$

Which, in the case of acetophenone, is:

$$Yield = \frac{1.22}{3} \times 68.5\% = 27.9\ \%$$

Supplementary note 8: Gas chromatographs of the gas phase of the cumene oxidation reaction

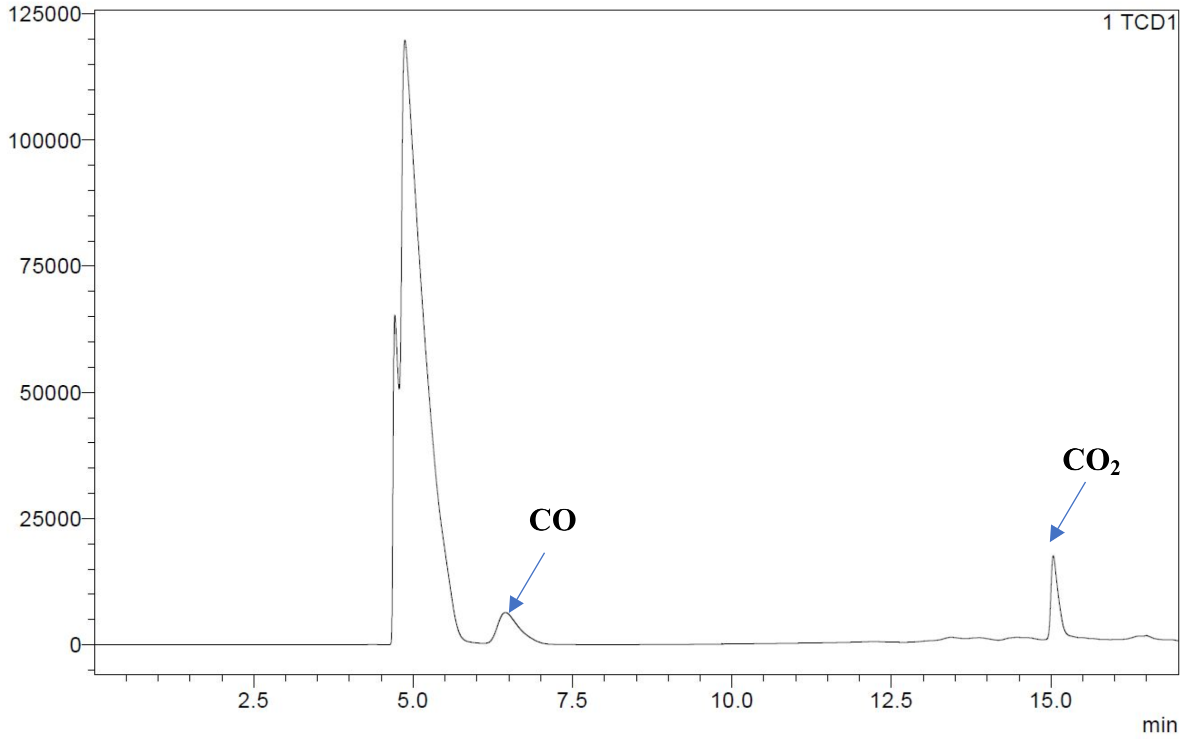

Fig. S8. Chromatograph of gas phase of the cumene oxidation reaction.

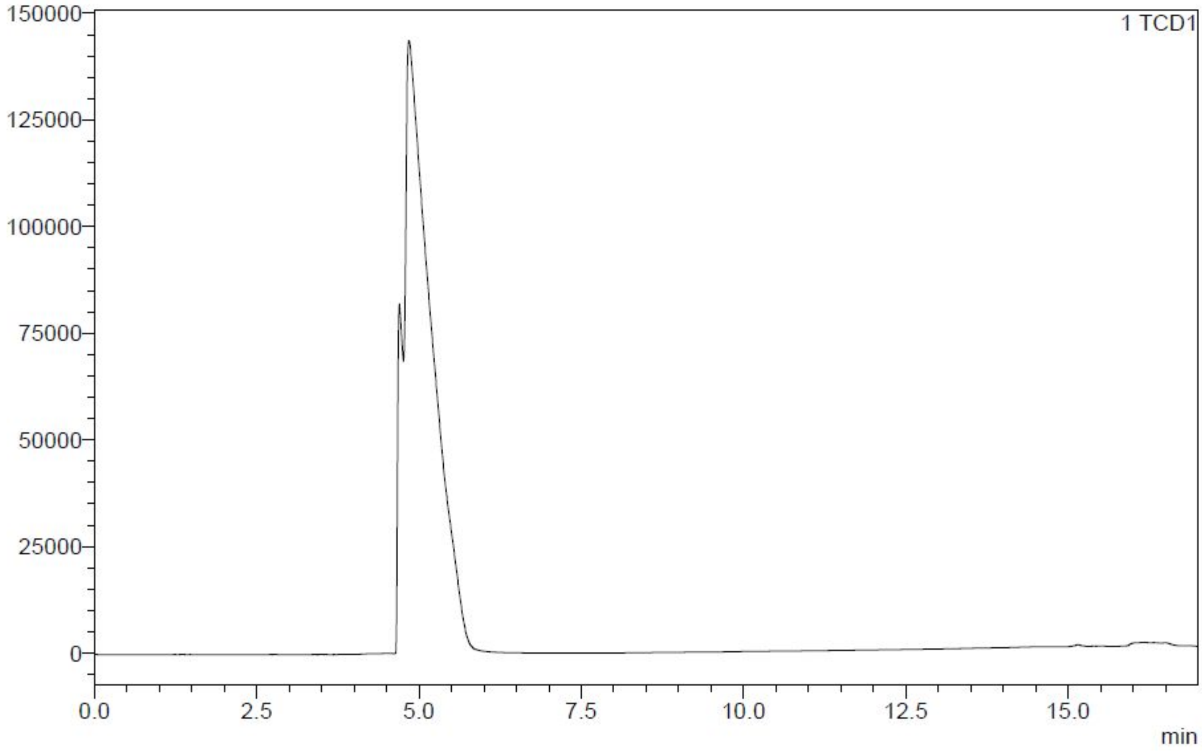

Fig. S9. Chromatograph of the ambient air.

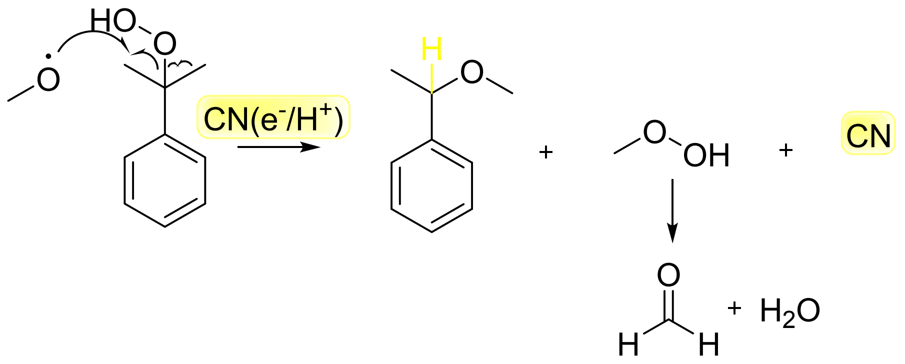

Fig. S10. Proposed mechanism for the synthesis of the 1-methoxyethylbenzene.

References

[1] G. Filippini, F. Longobardo, L. Forster, A. Criado, G. Di Carmine, L. Nasi, C. D'Agostino, M. Melchionna, P. Fornasiero, M. Prato, Light-driven, heterogeneous organocatalysts for C–C bond formation toward valuable perfluoroalkylated intermediates, Science Advances, 6 eabc9923.

[2] D. Baranowska, K. Zielinkiewicz, E. Mijowska, B. Zielinska, Sugars induced exfoliation of porous graphitic carbon nitride for efficient hydrogen evolution in photocatalytic water-splitting reaction, Scientific Reports, 14 (2024) 1998.

[3] H.-J. Li, D.-J. Qian, M. Chen, Templateless Infrared Heating Process for Fabricating Carbon Nitride Nanorods with Efficient Photocatalytic H2 Evolution, ACS Applied Materials & Interfaces, 7 (2015) 25162-25170.

[4] M.B. Idris, G.H. Sai, D. Hemalatha, G. Sakthivel, S. Devaraj, The Effect of Phosphorous Doping on the Composition and Capacitance Properties of Mesoporous Graphitic Carbon Nitride, Journal of The Electrochemical Society, 166 (2019) A2409.

[5] M. Fronczak, M. Krajewska, K. Demby, M. Bystrzejewski, Extraordinary Adsorption of Methyl Blue onto Sodium-Doped Graphitic Carbon Nitride, The Journal of Physical Chemistry C, 121 (2017) 15756-15766.

[6] A. Khan, U. Alam, S. Zafar, M. Muneer, Fe(III)-grafted K-doped $$\hbox {g-C}_{{3}}\hbox {N}_{{4}}$$/rGO composite photocatalyst with efficient activity towards the degradation of organic pollutants$$^{\S }$$, Journal of Chemical Sciences, 130 (2018) 142.

[7] S. Neamani, L. Moradi, Loading of g-C3N4 on Core-Shell Magnetic Mesoporous Silica Nanospheres as a Solid Base Catalyst for the Green Synthesis of some Chromene Derivatives under Different Conditions, ChemistryOpen, 11 (2022) e202200041.

[8] Y. Wang, X. Wang, M. Antonietti, Polymeric Graphitic Carbon Nitride as a Heterogeneous Organocatalyst: From Photochemistry to Multipurpose Catalysis to Sustainable Chemistry, Angew. Chem. Int. Ed., 51 (2012) 68-89.

[9] D. Cruz, S. Żółtowska, O. Savateev, M. Antonietti, P. Giusto, Carbon nitride caught in the act of artificial photosynthesis, Nature Communications, 16 (2025) 374.

[10] N. Hellgren, R.T. Haasch, S. Schmidt, L. Hultman, I. Petrov, Interpretation of X-ray photoelectron spectra of carbon-nitride thin films: New insights from in situ XPS, Carbon, 108 (2016) 242-252.

[11] J. Tong, L. Zhang, F. Li, M. Li, S. Cao, An efficient top-down approach for the fabrication of large-aspect-ratio g-C3N4 nanosheets with enhanced photocatalytic activities, Physical Chemistry Chemical Physics, 17 (2015) 23532-23537.

[12] M. Quaranta, M. Murkovic, I. Klimant, A new method to measure oxygen solubility in organic solvents through optical oxygen sensing, Analyst, 138 (2013) 6243-6245.

[13] Q. Yan, C. Zhao, L. Zhang, Y. Hou, S. Wang, P. Dong, F. Lin, Y. Wang, Facile Two-Step Synthesis of Porous Carbon Nitride with Enhanced Photocatalytic Activity Using a Soft Template, ACS Sustainable Chemistry & Engineering, 7 (2019) 3866-3874.

[14] Y. Jin, D. Zheng, Z. Fang, Z. Pan, S. Wang, Y. Hou, O. Savateev, Y. Zhang, G. Zhang, Salt-melt synthesis of poly(heptazine imide) in binary alkali metal bromides for enhanced visible-light photocatalytic hydrogen production, Interdisciplinary Materials, 3 (2024) 389-399.

[15] M. Chang, Z. Pan, D. Zheng, S. Wang, G. Zhang, M. Anpo, X. Wang, Salt-Melt Synthesis of Poly Heptazine Imides with Enhanced Optical Absorption for Photocatalytic Hydrogen Production, ChemSusChem, 16 (2023) e202202255.

[16] K. Murugesan, A. Sagadevan, L. Peng, O. Savateev, M. Rueping, Recyclable Mesoporous Graphitic Carbon Nitride Catalysts for the Sustainable Photoredox Catalyzed Synthesis of Carbonyl Compounds, ACS Catalysis, 13 (2023) 13414-13422.

[17] L. Schmermund, S. Reischauer, S. Bierbaumer, C.K. Winkler, A. Diaz-Rodriguez, L.J. Edwards, S. Kara, T. Mielke, J. Cartwright, G. Grogan, B. Pieber, W. Kroutil, Chromoselective Photocatalysis Enables Stereocomplementary Biocatalytic Pathways, Angewandte Chemie International Edition, 60 (2021) 6965-6969.
[18] Y. Zhong, H. Zhu, X. Xie, L. Yang, Y. Shen, Q. Fan, Z. Xie, Z. Le, All-Solid-State Z-Scheme CsPbBr3/Au/g-C3N4 Heterojunctions for Enhanced Photocatalytic C–H Oxidation, Inorganic Chemistry, 64 (2025) 2706-2715.
[19] P. Chen, Y. Li, C. Xiao, L. Chen, J.-K. Guo, S. Shen, C.-T. Au, S.-F. Yin, Preparation of Helical BiVO4/Ag/C3N4 for Selective Oxidation of C–H Bond under Visible Light Irradiation, ACS Sustainable Chemistry & Engineering, 7 (2019) 17500-17506.
[20] P. Zhang, Y. Wang, J. Yao, C. Wang, C. Yan, M. Antonietti, H. Li, Visible-Light-Induced Metal-Free Allylic Oxidation Utilizing a Coupled Photocatalytic System of g-C3N4 and N-Hydroxy Compounds, Advanced Synthesis & Catalysis, 353 (2011) 1447-1451.
[21] S. Verma, R.B. Nasir Baig, M.N. Nadagouda, R.S. Varma, Photocatalytic C–H Activation of Hydrocarbons over VO@g-C3N4, ACS Sustainable Chemistry & Engineering, 4 (2016) 2333-2336.
[22] S. Bishi, B. Sankar Lenka, P. Kreitmeier, O. Reiser, D. Sarkar, g-C3N4 Photocatalyzed Decarboxylative Oxidation of Carboxylic Acids and the Oxidation of Alkenes and Alkanes, Advanced Synthesis & Catalysis, 366 (2024) 3397-3403.
[23] R.P. Gaikwad, I.R. Warkad, D.S. Chaudhari, H.N. Pham, A.K. Datye, M.B. Gawande, Nanoarchitectonics of vanadium nanoparticles decorated mesoporous carbon nitride in photocatalytic systems: A study on ethylbenzene oxidation reaction, Journal of Colloid and Interface Science, 679 (2025) 54-63.
[24] C. Wang, Y. Hou, J. Cheng, M.-J. Lin, X. Wang, Biomimetic donor-acceptor motifs in carbon nitrides: Enhancing red-light photocatalytic selective oxidation by rational surface engineering, Applied Catalysis B: Environmental, 294 (2021) 120259.
[25] S. Tang, Z. Fu, Y. Li, Y. Li, Study on boron and fluorine-doped C3N4 as a solid activator for cyclohexane oxidation with H2O2 catalyzed by 8-quinolinolato ironIII complexes under visible light irradiation, Applied Catalysis A: General, 590 (2020) 117342.
[26] J. Li, S. Zhao, S.-Z. Yang, S. Wang, H. Sun, S.P. Jiang, B. Johannessen, S. Liu, Atomically dispersed cobalt on graphitic carbon nitride as a robust catalyst for selective oxidation of ethylbenzene by peroxymonosulfate, Journal of Materials Chemistry A, 9 (2021) 3029-3035.
[27] H. Zhu, J. Zhao, C. Ma, Z. Yu, J. Li, Q. Meng, Bridging Effect of Carbon Nitride with More Negative Conduction Potential and Halogens Promotes the Liquid-Phase Oxidation of Aromatic C–H Bonds, ACS Applied Materials & Interfaces, 15 (2023) 59280-59295.
[28] B. Mühldorf, R. Wolf, Photocatalytic benzylic C–H bond oxidation with a flavin scandium complex, Chemical Communications, 51 (2015) 8425-8428.
[29] H. Zhu, J. Zhao, Z. Yu, J. Li, C. Ma, H. Sun, Y. Wu, Q. Meng, Visible Light-Driven Sandwich-like g-C3N4-Catalyzed Oxidation to Produce Cumene Hydroperoxide, Industrial & Engineering Chemistry Research, 62 (2023) 8253-8268.
[30] P. Geng, Y. Tang, G. Pan, W. Wang, J. Hu, Y. Cai, A g-C3N4-based heterogeneous photocatalyst for visible light mediated aerobic benzylic C–H oxygenations, Green Chemistry, 21 (2019) 6116-6122.
[31] A.P. Shah, A.S. Sharma, V.S. Sharma, N.G. Shimpi, Polyacrylonitrile Nanofibers Incorporating Silver-Decorated Graphitic Carbon Nitride for the Visible-Light-Activated Selective Oxidation of Styrene, Benzylic Methylene Groups, and Benzene, ACS Applied Nano Materials, 3 (2020) 1922-1933.
[32] H. Zhu, J. Zhao, J. Li, C. Ma, Z. Yu, Q. Meng, Visible-light-driven g-C3N4-doped Co catalyzed oxidation of benzylic hydroxylation of alkyl aromatic hydrocarbons, Chemical Engineering Science, 267 (2023) 118365.

[33] C.A. Brown, T.A. Nile, M.F. Mahon, R.L. Webster, Iron catalysed Negishi cross-coupling using simple ethyl-monophosphines, Dalton Transactions, 44 (2015) 12189-12195.
[34] T. Fukuyama, Y. Fujita, H. Miyoshi, I. Ryu, S.-C. Kao, Y.-K. Wu, Electron transfer-induced reduction of organic halides with amines, Chemical Communications, 54 (2018) 5582-5585.
